# *Cecidonius pampeanus*, gen. et sp. n.: an overlooked and rare, new gall-inducing micromoth associated with *Schinus* in southern Brazil (Lepidoptera, Cecidosidae)

**DOI:** 10.3897/zookeys.695.13320

**Published:** 2017-09-04

**Authors:** Gilson R.P. Moreira, Rodrigo P. Eltz, Ramoim B. Pase, Gabriela T. Silva, Sérgio A.L. Bordignon, Wolfram Mey, Gislene L. Gonçalves

**Affiliations:** 1 Departamento de Zoologia, Instituto de Biociências, Universidade Federal do Rio Grande do Sul, Av. Bento Gonçalves 9500, 91501-970 Porto Alegre, RS, Brazil; 2 PPG Biologia Animal, Departamento de Zoologia, Instituto de Biociências, Universidade Federal do Rio Grande do Sul, Av. Bento Gonçalves 9500, 91501-970 Porto Alegre, RS, Brazil; 3 Programa de Mestrado em Avaliação de Impactos Ambientais, Universidade La Salle, Av. Victor Barreto, 2288, 92010-000 Canoas, RS, Brazil; 4 Museum für Naturkunde, Leibniz Institute for Evolution and Biodiversity Science, Invalidenstraße 43, 10115 Berlin, Germany; 5 PPG Genética e Biologia Molecular, Departamento de Genética, Instituto de Biociências, Universidade Federal do Rio Grande do Sul, Av. Bento Gonçalves 9500, 91501-970 Porto Alegre, RS, Brazil; 6 Departamento de Recursos Ambientales, Facultad de Ciencias Agronómicas, Universidad de Tarapacá, Casilla 6-D, Arica, Chile

**Keywords:** Anacardiaceae, cecidosid moths, conservation, insect galls, Neotropical region, taxonomy

## Abstract

Galls induced by the larval stage of cecidosids (Lepidoptera: Cecidosidae) are complex, multi-trophic systems, still poorly studied. They may be associated with other insect feeding guilds, including inquilines, kleptoparasites, cecidophages, parasitoids, and predators. By causing death of the gall inducer early in life and altering the gall phenotype, inquilines may lead to misidentification of the true gall inducers. Here, we describe through light and scanning electron microscopy *Cecidonius pampeanus*, a new genus and species of cecidosid moth, from the Pampa biome, south Brazil. It induces unnoticed, small galls under swollen stems of *Schinus weinmannifolius* Mart. ex Engl. (Anacardiaceae). Such galls are severely attacked early in ontogeny either by unidentified parasitoids belonging to *Lyrcus* Walker (Pteromalidae) that feed upon the inducer, or by inquiline wasps of the genus *Allorhogas* Gahan (Braconidae). The inquilines modify the galls into large ones that last longer and promptly call attention. Free-living galls are rare and dehiscent, pupation of *C. pampeanus* occurring on the ground. Due to these reasons the true inducer has been overlooked in this case for more than a century. Additionally we inferred a phylogeny for Cecidosidae using sequences from mitochondrial and nuclear loci, and characterized genetic variation and gene flow across ten populations. Despite its natural history similarities with the African genus *Scyrotis*, *Cecidonius* is a much younger lineage, more closely related to the Neotropical cecidosids. *C. pampeanus* populations, which are now confined to a few mountain areas within its distribution range due to habitat destruction, are also genetically isolated, requiring conservation measures.

## Introduction

Insect-induced galls may consist of very complex, multitrophic-level systems including not only the gall inducers themselves, but also predators, cecidophages, parasitoids, kleptoparasites and inquilines, among other insects such as successors that use them for shelter. Kleptoparasites in particular invade galls, usurping the cecidogenous species and become stationary, feeding upon gall tissues until they complete their larval development, and may prey upon the inducer and other insects that eventually enter the usurped gall (e.g., Morris et al. 2000, Luz et al. 2015). They do not induce differentiation and growth of new tissues, only feeding on those that were induced to develop by their precursors. Inquilines, however, induce the development of new tissues, either similar to or different from original ones when they take over a given gall, generally killing the inducer by inanition (e.g., Brooks and Shorthouse 1988, van Noort et al. 2007). Thus, they may change substantially the size and shape of the gall they invade. Little attention has been paid to the important taxonomic consequences of this phenomenon, a potential difficulty factor in identification of hidden diversity in gall communities. Misidentification of the true gall inducers in such cases is obviously likely, since the inducer is eliminated from the system early in the gall ontogeny and no conspicuous trace of it may be left inside the gall. In addition, contrary to galls attacked either by kleptoparasites or inquilines that may stay attached to host plants, those free of them still containing the growing inducer may be dehiscent, with later development of immature stages occurring on the ground (e.g., van Noort et al. 2007, Luz et al. 2015). In this case, by altering the place of gall development in the field and thus enhancing the encounter of attacked galls by kleptoparasites and inquilines that stay attached to the host plant compared to free, detached ones, the possibility of missing the presence of the true inducers is substantially increased. Furthermore, depending on the rate of attack by other parasitoids and predators in association, natural densities of the true gall inducer would be reduced further, even becoming rare, and thus may be unnoticed. As a case study, here we describe one example of such a peculiar system, where the induction of a non-conspicuous, dehiscent gall by a cecidosid moth has been overlooked for more than a century, erroneously believed to be induced by their hymenopteran inquilines who do not originally induce galls but in fact modify them early in development into large and colorful, visually appealing galls.

Cecidosidae are poorly known monotrysian Heteroneura moths (*sensu* Davis 1998), comprising six genera and 18 species, all with ranges restricted to the southern hemisphere. They are among a few lepidopteran lineages with a Gondwanic distribution: one occurs in New Zealand, the monotypic genus *Xanadoses* Hoare & Dugdale; twelve in southern Africa, all belonging to *Scyrotis* Meyrick, and five in South America, two in *Dicranoses* Kieffer & Jörgensen, and three in the monotypic genera *Cecidoses* Curtis, *Eucecidoses* Brèthes, and *Oliera* Brèthes. *Xanadoses nielseni* Hoare & Dugdale is a bark-miner of several New Zealand bark trees, particularly within *Weinmannia* Linnaeus (Cunoniaceae). Larvae of African *Scyrotis* form galls on species of *Searsia* F.A. Barkley (Anacardiaceae) (van Noort et al. 2007). In this case, they may also be located in the leaves; these galls are known as “jumping-beans”. They exfoliate from the hostplant and drop to the ground, where they are propelled for short distances by the active pupa inside, a supposed adaptation to avoid excessive heat from the sun (Meyrick 1917, Davis 1998). Unfortunately, none of the immature stages of *Scyrotis* species have been described in detail yet. South American cecidosids induce galls either on the stem or on axillary buds of *Schinus* Linnaeus (Anacardiaceae), particularly *S. polygamus* (Cav.) Cabrera (*sensu* Cabrera 1938, Fleig 1987, 1989). Gall morphology and life history of *C. eremita* Curtis have been treated in detail by Wille (1926). The taxonomy was reviewed and immature stages and galls of *O. argentinana* Brèthès, and *D. capsulifex* Kieffer & Jörgensen were described respectively by Moreira et al. (2012) and San Blas and Davis (2013). Information gathered recently by the first author suggested that diversity of cecidosids is much greater in the Neotropics, and not only additional species of *Schinus* are used as host but also other Anacardiaceae, such as species of *Lithraea* Miers ex Hook. & Arn.

This study concerns the galls of *Schinus weinmannifolius* Mart. ex Engl., which are induced by an undescribed genus and species of Cecidosidae in southern Brazil. Although not fully explored yet, the existence of these galls has been known for a long time; their induction was wrongly associated with cynipid wasps (Tavares 1909, Wille 1926, Houard 1933, Sáiz and Núñez 1997). Here the gall, the immature stages, and adults of the true inducer are described under both light and scanning electron microscopy and provided information on its natural history, in conjunction with a parasitoid and an inquiline wasp frequently found in association with these galls. By conducting an analysis of concatenated mitochondrial (COI and 16S) and nuclear (Wingless) DNA sequences including putative members of all known Neotropical cecidosid lineages, we provide further support for the proposition of the new taxon. Considering the possibility that the new species could be closely related to the African lineages, two *Scyrotis* species are also included for the first time for comparison in the phylogenetic analysis of Cecidosidae. Given that extant populations of the new taxa are in low numbers and restricted to a reduced distribution range, a genetic structure analysis was carried out using *ca.* 1.5 kb of COI gene sequences. Statistical analysis was performed to describe the genetic diversity of this rare species. Data are discussed in the context of importance regarding use of integrative taxonomy, including molecular analyses, in the discovery of hidden insect diversity in gall communities and the corresponding conservation scenario.

## Materials and methods

### Morphology

Adult specimens used in this study were reared from galls in small plastic vials, which were maintained under controlled conditions (14 h light/10 h dark; 25 ± 2 °C) in the Laboratório de Morfologia e Comportamento de Insetos (**LMCI**), Departamento de Zoologia, Universidade Federal do Rio Grande do Sul (**UFRGS**), Porto Alegre city, RS. Dehiscent galls (approx. 20 in total) were collected from the ground, in the surroundings of *S. weinmannifolius* plants of an old grass field, located in a farm belonging to Antonio Malta, Coxilha das Lombas, 30°01'46"S, 50°36'40"W, 86m, 29.V.2012, Santo Antônio da Patrulha Municipality, Rio Grande do Sul State (RS), Brazil. Pupae were obtained later (September) by dissecting some galls under a stereomicroscope in the laboratory. Larvae were obtained by dissecting *S. weinmannifolius* branches, either from galls located under swollen bark (early instars) or erupted from the stem (later instars). Adults were pin-mounted and dried. Immature stages were fixed in Dietrich´s fluid and preserved in 75% ethanol. Larvae used for DNA extraction came from several additional populations (listed below), and were preserved in 100% ethanol at -20 °C.

For descriptions of adult morphology the specimens were cleared in a 10% potassium hydroxide (KOH) solution, stained with Chlorazol black E and slide-mounted in either glycerine jelly or Canada balsam. Last instar larvae were prepared similarly for description of chaetotaxy. Observations were performed with the aid of a Leica® M125 stereomicroscope. Structures selected to be drawn were previously photographed with a Sony® Cyber-shot DSC-H10 digital camera attached to the stereomicroscope. Vectorised line drawings were then made with the software Corel Photo-Paint® X7, using the corresponding digitalized images as a guide. Additional specimens were used for scanning electron microscope analyses. They were dehydrated in a Bal-tec® CPD030 critical-point dryer, mounted with double-sided tape on metal stubs, coated with gold in a Bal-tec® SCD050 sputter coater and examined and photographed in a JEOL® JSM6060 scanning electron microscope at the Centro de Microscopia Eletrônica (CME) of UFRGS.

### Molecular analysis

Mitochondrial and nuclear DNA sequences were used for two different levels of analysis of the undescribed genus and species: 1) to infer the phylogenetic status and relationships within Cecidosidae, and 2) to describe the genetic diversity and population structure of this rare taxon. For the first approach we used representative species of all members of Cecidosidae except *Xanadoses*, the corresponding samples coming from the tissue collection of LMCI: i.e., *C. eremita*, *Dicranoses congregatella* Kieffer & Jörgensen, *Eucecidoses minutanus* Brèthes, *O. argentinana*, an undescribed lineage from Chile (previously known to be closely related based on morphology) and *Scyrotis* (*Scyrotis* sp. and *S. granosa* Meyrick), a genus from Africa included for the first time in a molecular phylogeny. For the second approach we sampled 10 populations across the distribution range of *Cecidonius pampeanus* sp. n. (P1 to P10), including six individuals per site (Suppl. materials 3, 5). Previous analyses indicated there was no substantial addition of genetic variation by increasing corresponding sample size. Total genomic DNA was purified from fresh collected larval tissue of all Cecidosidae surveyed except *Scyrotis* (dried museum adult specimens were used), using the PureLink genomic DNA kit (Life, Invitrogen, USA) following the manufacturer’s instructions.

For cecidosid phylogeny we used nucleotide sequences obtained from different molecular markers, selected because they evolve at different rates and provide phylogenetic resolution at different, overlapping taxonomic levels: two mitochondrial (1421 bp of the cytochrome oxidase subunit I [COI] and 474 bp of the 16S ribosomal RNA [16S] genes), and one nuclear (395 bp of the Wingless [Wg] gene) loci. For the genetic structure and variability approach, we amplified COI in 60 individuals, six from each population sampled. The selected molecular markers were amplified by polymerase chain reaction (PCR); primers and conditions used are described in the supplementary material (Suppl. material 1). PCR products were purified using the enzymatic method (exonuclease and alkaline phosphatase), sequenced with BigDye chemistry, and analysed in an ABI3730XL (Applied Biosystems Inc.). Chromatograms obtained from the automatic sequencer were read and sequences were assembled using the software CodonCode Aligner (CodonCode Corporation). Sequences generated in this study were deposited in the GenBank database (Suppl. material 3).

Sequence data were used for the reconstruction of a concatenated phylogenetic tree (COI+16S+Wg) with the Bayesian method in BEAST 2.02 (Bouckaert et al. 2014). The tree prior was set as a Yule calibrated process, using GTR + I for the COI partition and TN92+G for both 16S and Wg, selected with the Bayesian information criterion (BIC; Schwarz 1978) for each data set in jModelTest 2.1.2 (Darriba et al. 2012). The branch lengths were allowed to vary under a relaxed clock model with an uncorrelated log-normal distribution (Drummond et al. 2006). To adjust the molecular clock we used the fossil calibration point of Adeloidea (sensu van Nieukerken et al. 2011), about 120±10mya, with a log-normal distribution (Walhberg et al. 2013). The analysis was run for 10,000,000 Markov Chain Monte Carlo (MCMC) cycles and parameters were sampled every 1,000 cycles; this was repeated four times to test for MCMC convergence, and priors exceeded 200 to ensure effective sample sizes (ESS). Burn-in was determined in Tracer 1.5 (Drummond and Rambaut 2007) based on ESS and parameter trajectories, and the first 20% of trees were then removed with TreeAnnotator. Trees were observed and edited in FigTree v1.3.1 (Rambaut 2009). Clades with Bayesian Posterior Probability (BPP) ≥ 95% were considered strongly supported. Pairwise genetic distances (p-distances) among lineages were calculated in MEGA 7 (Tamura et al. 2013).

Nucleotide and haplotype (gene) diversity indices were estimated for individuals grouped into ten populations (P1 to P10) with DnaSP 5.1 (Librado and Rozas 2009). To investigate the evolutionary relationships among COI-haplotypes a median-joining haplotype network (Bandelt et al.1999) was constructed in NETWORK 5 (http://www.fluxus-engineering.com/sharenet.htm). Levels of genetic structure among populations were characterized using φST with Arlequin3.5 (Excoffier and Lischer 2010). Additionally, to investigate spatial patterns of genetic structure we assessed the correlation between genetic and geographic distances for all pairs of sampled individuals using a Mantel test (Mantel 1967). We also performed an Analysis of Molecular Variance (AMOVA; Excoffier et al. 1992) with Arlequin to assess more detailed quantitative differentiation among populations, performing two rounds of AMOVA employing different geographic clustering strategies: i) taking into account the vicariate effect of the Jacui River, and ii) using the genetic distance and haplotype relationship results. Finally, we investigated the demographic history of the new genus and species using neutrality tests (Tajima’s D, Fu & Li’ D* and F*, Fu’s Fs) and mismatch distribution analysis (Rogers and Harpending 1992, Schneider and Excoffier 1999) with DnaSP.

Abbreviations of the institutions from which specimens were examined are:

**DZUP** Coll. Padre Jesus S. Moure, Departamento de Zoologia, Universidade Federal do Paraná, Curitiba, Paraná, Brazil.

**LMCI** Laboratório de Morfologia e Comportamento de Insetos, Universidade Federal do Rio Grande do Sul, Porto Alegre, Rio Grande do Sul, Brazil.

## Results

### Molecular phylogeny

**Figure 1.**
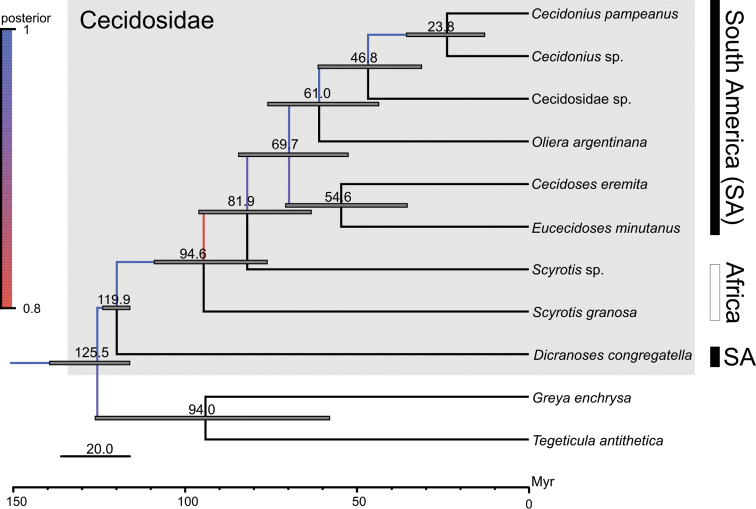
Molecular phylogeny of Cecidosidae. Bayesian time-calibrated consensus tree based on cytochrome oxidase subunit I (COI), r16S ribosomal (16S) and Wingless (Wg) genes. Prodoxidae (*Greya enchrysa* and *Tegeticula antithetica*) was used to root the tree. Colored branches indicate posterior probability support for the equivalent node following the legend. Dark gray bar indicates confidence interval for each node age estimate, presented in millions of years ago (Mya).

The phylogenetic reconstruction corroborated our hypothesis of monophyly (well supported by posterior probability) for the new proposed genus (Fig. 1). Its sister taxon is the undescribed lineage from Chile (Cecidosidae sp.); it was close to *O. argentinana* among the described species of cecidosids. The dated phylogeny revealed the new genus as the youngest lineage among cecidosids, which emerged around 23.8 Mya (95% HDP 10.2–34.6 mya). Genetic distances of the new genus to other cecidosids ranged from 9 to 25%; less divergence was observed in relation to the sister species *Cecidonius* sp. and highest to *D. congregatella* (Table 1).

**Table 1.** Estimates of pairwise genetic distance (%) among nine Cecidosidae lineages based on DNA sequences (1420 base pairs of the cytochrome oxidase subunit I gene) using p-distance.

	*Cecidonius pampeanus*	*Cecidonius* sp.	*Cecidoses eremita*	*Dicranoses congregatella*	*Eucecidoses minutanus*	*Cecidosidae* sp.	*Oliera argentinana*	*Scyrotis* sp.	*Scyrotis granosa*
*Cecidonius pampeanus*	–								
*Cecidonius* sp.	9.0	–							
*Cecidoses eremita*	18.3	20.7	–						
*Dicranoses congregatella*	25.0	25.2	25.4	–					
*Eucecidoses minutanus*	13.8	18.6	18.3	23.2	–				
Cecidosidae sp.	16.1	18.3	18.5	23.7	17.8	–			
*Oliera argentinana*	13.7	16.4	17.5	21.8	16.3	15.1	–		
*Scyrotis* sp.	19.4	20.8	21.3	25.0	21.4	20.3	18.2	–	
*Scyrotis granosa*	23.7	27.4	26.9	28.8	27.8	25.8	24.5	27.3	–

### Taxonomy

#### Cecidonius

Taxon classificationAnimaliaLepidopteraCecidosidae

Moreira & Gonçalves gen. n.

http://zoobank.org/5029391A-325F-4BB4-A726-8D5F9FB78476

Figs 2 , 3 , 4 , 5 , 6 , 7 , 8 , 9

##### Type species.

*Cecidonius pampeanus* Moreira & Gonçalves, new species

##### Diagnosis.

*Cecidonius* gen. n. bears several adult, pupal, larval, and gall features that in conjunction differentiate it from all cecidosid genera. Unlike other cecidosids, adults of *Cecidonius* have lateral cervical sclerites with anterior arms short and posterior ones with distal portion membranous. Females have a long ovipositor, bearing a large oviscapt cone with internal dorsal crest that extends cephalad within the seventh abdominal segment. In particular, they differ from those of the New Zealand *Xanadoses* that have a well-developed proboscis and five-segmented maxillary palpus (Hoare and Dugdale 2003) by having a vestigial proboscis and two-segmented maxillary palpus, among other characters. Unlike all species of the African *Scyrotis* that have forewings with four radial veins (Mey 2007), *Cecidonius* has five R-veins. With the exception of *Oliera*, which has small rudiments of galea (Moreira et al. 2012), other South American cecidosids show no vestiges of such structures. However, adults of *Cecidoniu*s have moderately well-developed galea. Contrary to those of *Oliera* where the maxillary and labial palpi are respectively three- and two-segmented, *Cecidonius* has the reverse; that is, two- and three-segmented maxillary and labial palpi, respectively. The pupa of *Cecidonius* is unique among all described cecidosids (those of *Scyrotis* are unknown), by having a stout and truncate cocoon cutter, flanked at the base by a pair of small, similarly shaped processes. In addition, in *Cecidonius* pupae the anterior margin of abdominal terga bear strong, posteriorly directed, transversally aligned spines that are much smaller in other genera. The larva of *Cecidonius* is also unique in having long thoracic setae, compared to short abdominal ones. They have two pair of stemmata; there is one in *Xanadoses*, and they are absent in other South American genera (larvae of *Scyrotis* are also unknown). Their woody, cylindrical galls are also unique, initially developing within swollen stems of *S. weinmannifolius* in southern Brazil. Later in ontogeny, they rupture the plant stem, thus growing externally. They are dehiscent, falling to the ground where pupation occurs. Contrary to those of *Scyrotis* (for detail, see von Noort et al. 2007), they do not exfoliate from the stem; they detach with their proximal base open, the corresponding orifice being clogged by larval feces.

##### Description of adults

**Figure 2.**
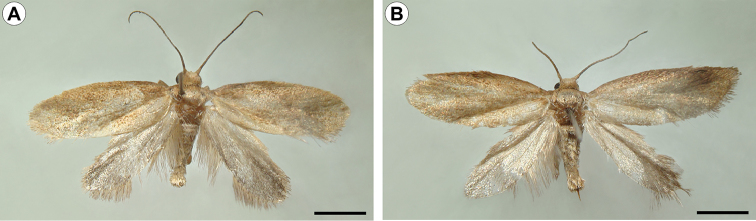
Pinned-dried *C. pampeanus* adults, dorsal view: **A** male (holotype, LMCI 188-4) **B** female paratype (LMCI 188-6). Scale bars: 2 mm.

**Figure 3.**
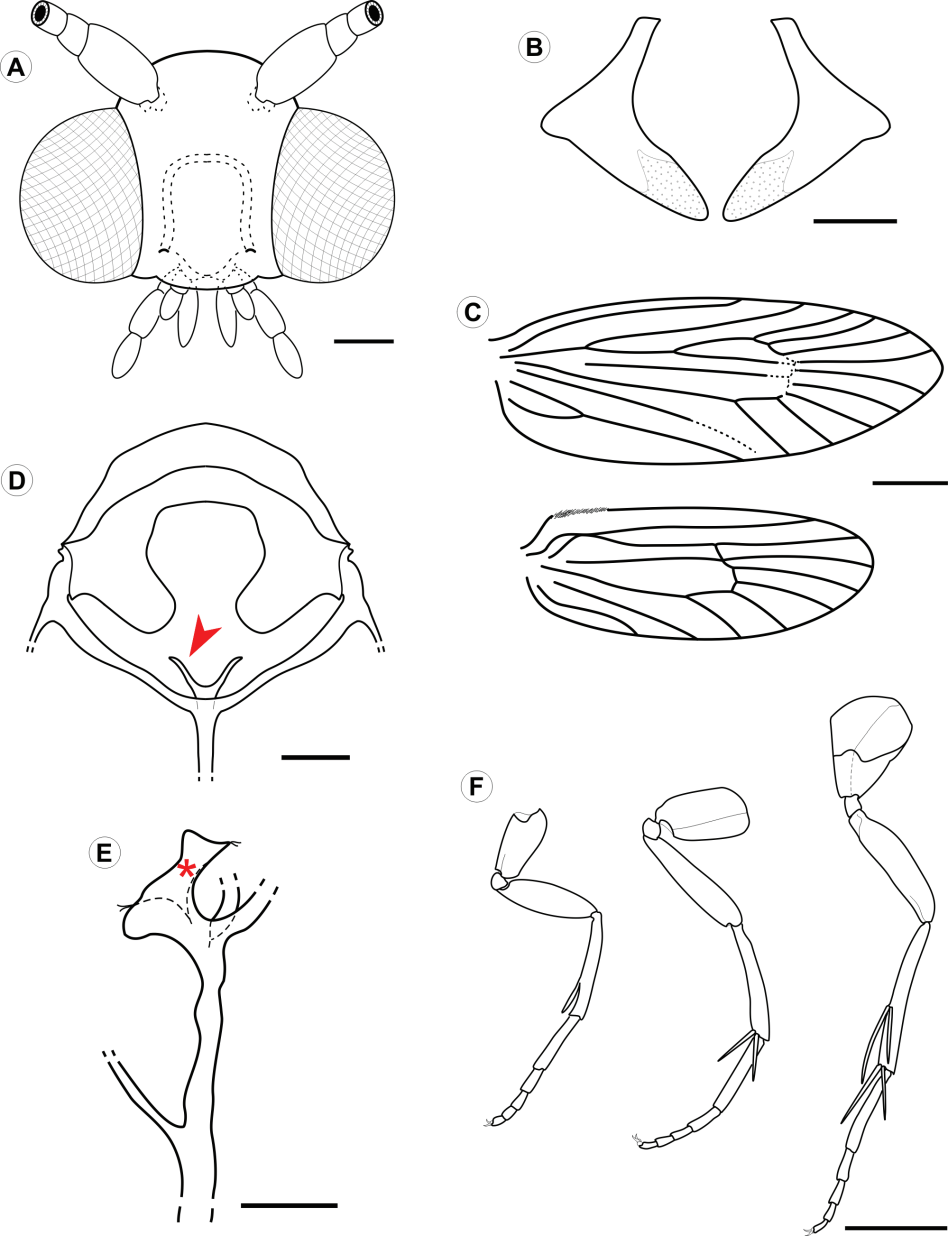
*Cecidonius pampeanus* adult morphology under light microscopy. **A** head, anterior view **B** lateral cervical sclerites, anterior; **C** fore- and hindwing venation, dorsal **D** metathoracic furcasternum, posterior (closed arrow points to left furcal apophysis) **E** metathoracic furcasternum in detail, lateral (asterisk indicates left furcal apophysis) **F** fore-, median- and hindlegs, from left to right, respectively. Scale bars: 0.25 (**A, D**); 0.1 mm (**B**); 1 mm (**C, F**); 0.2 mm (**E**).

**Figure 4.**
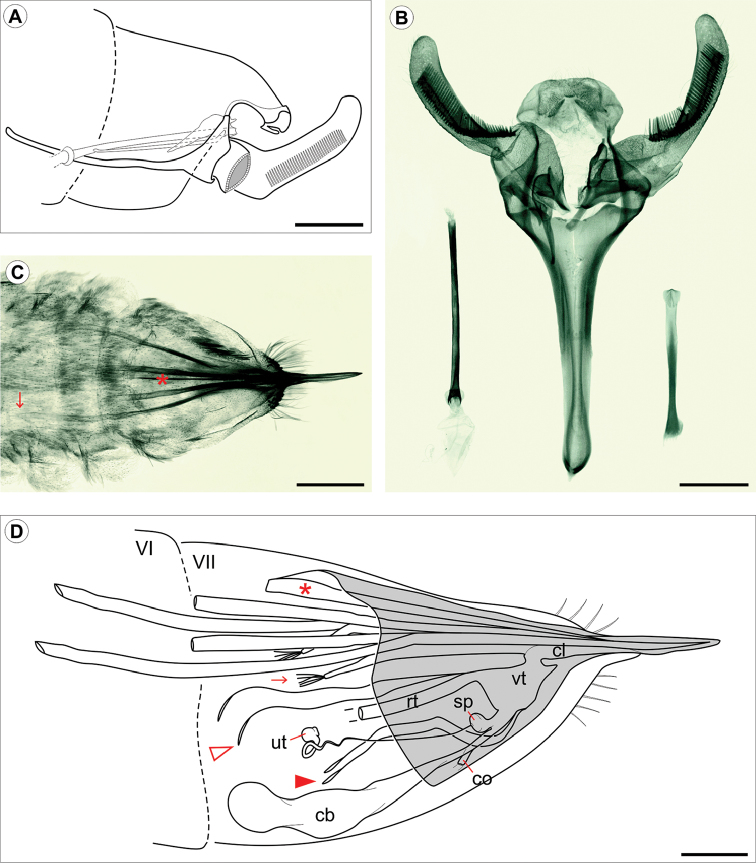
*Cecidonius pampeanus* genitalia morphology under light microscopy. **A** schematic representation of male genitalia, lateral view (left valve omitted) **B** dissected male genitalia, ventral, with detached phallus and juxta, on left and right side, respectively **C** female genitalia, dorsal **D** schematic representation of female genitalia, latero-dorsal. Roman numbers indicate abdominal segments. Oviscapt cone is represented in light gray in **D**. Arrows point to the end of left anterior apophysis in **C**, and to the apodeme of posterior apophysis in **D**. Asterisks indicate internal dorsal crest of oviscapt cone in **C** and **D**. Open and closed arrow heads point, respectively, to posterior apophyses and cloacal apodemes in **D**. Abbreviations: **cb** corpus bursae; **cl** cloaca; **co** common oviduct; **sp** spermatheca; **rt** rectum; **vt** vestibulum; **ut** utriculus of spermatheca. Scale bars: 0.25 mm.

(Figs 2–4). Male and female similar in size and color; the body is covered with uniform, faded copper-coloured scales. Small moth, forewing length 4.16–4.58 mm (n = 4). *Head* (Fig. 3A): frons and vertex smooth, with sutures weakly developed; vestiture consisting of a pair of latero-dorsal scale tufts curved forward over the frons. Scales slender, lamellar, suberect and scattered over labrum, haustellum, maxillary, and labial palpi. Eyes relatively large, rounded; vertical diameter ~ 2.0x, minimum interocular distance across frons. Antennae median (~ 0.7x length of forewing); scape smooth except for medium dense pecten; flagellum filiform, with slender scales scattered only over dorsal half; ventral half with several elongate sensilla *ca.* 0.7× length of flagellomere. Labrum greatly reduced. Pilifers and mandibles absent. Haustellum moderately developed (~2/3 labial palpi length). Maxillary palpi short, 2-segmented; ratios of segments from base ~1.0:1.4. Labial palpi 3-segmented, bent anteriorly and upward (~2/3 eye width in length); ratio of segments from base ~1.0:1.8:1.6. *Thorax*: Anterior arms of laterocervical sclerites (Fig. 3B) short; posterior arms with distal portion weakly melanised. Metafurca (Fig. 3D, E) with slender, elongate postero-dorsal apophyses, free from secondary arms; antero-dorsal apodemes present. Wings (Fig. 3C) lanceolate; microtrichia reduced in number; accessory cell present; retinaculum absent. Wing coupling consisting of ~20 frenular scales arising in two to three irregular rows near base of costa. Veins 13 in number, all reaching the margin; L/W index ~2.9; Sc ending near midpoint of wing margin, radius with 5 free branches, M 3-branched, CuA 2-branched, CuA1 and M3 well separated from each other basally, CuP faint distally and not stalked with 1A+2A. Hindwing: ~0.8 forewing in length, L/W index ~2.9; Sc and R stalked and ending distally at midpoint of wing margin, Rs unbranched, M 3-branched, M1 and M2 well separated, CuA 2-branched, CuA1 and M3 well separated, CuP faded, not stalked with 1A+2A. Legs (Fig. 3F) with spurs 0-2-4; epiphysis present. Tibial length proportion (anterior / medium / posterior legs) ~ 0.6:0.7:1.0. *Abdomen*: Sternum 2 with broad, U-shaped caudal rim; tergosternal connection absent. Male with remaining pre-genital segments unmodified. Female with abdominal segment A7 ~ 4x the length of A6; caudal margin bearing a dense ring of stout, elongate setae.

***Male genitalia*** (Fig. 4A, B). Uncus moderately bilobed. Socii consisting of a pair of setigerous, dorsally directed lobes. Valva long and slender, with an elongate pectinifer along ventral margin extending ~ distal half-length of valva. Vinculum Y-shaped. Phallus (Fig. 4B) simple, slender, and tubular, rosette-like shaped anteriorly; vesica without cornuti. Juxta (Fig. 4B) elongate (~ 2/3 phallus length), slender, slightly spatulate distally and encircling phallus caudally. Saccus stout and tubular, ~ 1.3× length of valve.

***Female genitalia*** (Fig. 4C, D). Oviscapt cone (*sensu* Kristensen 2003, San Blas and Davis 2013) present, with internal dorsal crest long, reaching the anterior portion of tergum seven. Anterior apophyses long, extending beyond fifth abdominal segment. Posterior apophyses ~1.5× length of anterior apophyses, and with anteriorly attached apodemes of similar width. Posterior apophyses are caudally fused to form an acute ovipositor, whose apex is compressed and sagittate, the lateral ridge bearing minute serrations. A typical primitive monotrysian reproductive system, with cloaca and vestibulum each bearing a pair of slender apodemes that extend anteriorly within abdominal segment 7; vestibulum without sclerotized structures; ductus and corpus bursae membranous, the latter saculiform, without signum; spermatheca connected to small, saculiform utriculus by a slightly coiled, afferent canal.

##### Etymology.

The genus name is derived from a composition between the Portuguese *Cecidia* (a gall; from the Greek, *kekídion*) with *Don* (an English nickname). Thus, the generic name means “Don`s gall”, named after Donald Davis from the Smithsonian Institution, USA, in recognition of his great contribution to the development of world lepidopterology, and in particular for having kindly introduced the first author to the study of Neotropical cecidosids a few years ago. The name is to be treated as masculine.

#### Cecidonius pampeanus

Taxon classificationAnimaliaLepidopteraCecidosidae

Moreira & Gonçalves sp. n.

http://zoobank.org/15DA6F09-4BF7-45CD-B25A-26DF60EDC383

Figs 2 , 3 , 4 , 5 , 6 , 7 , 8 , 9

##### Diagnosis.

As discussed for the monotypic genus.

##### Description of adults.

As described for the monotypic genus.

##### Type material.

Brazil: Old grass field, private farm belonging to Antonio Malta, Coxilha das Lombas, 30°01'46"S, 50°36'40"W, 86m, Santo Antônio da Patrulha Municipality, Rio Grande do Sul State (RS), Brazil; G.R.P. Moreira, H. A.Vargas, R. Brito & S.A.L. Bordignon; 29.V.2012, pinned-dry preserved adults, reared by the first author from dehiscent galls collected on the ground around patches of *Schinus weinmannifolius* Mart. ex Engl. plants. Holotype ♂: LMCI 188-4, emerged on 9.XI.2012; donated to DZUP (33.342). Paratypes: 1♂ (LMCI 188-7), emerged on 21.XI.2012, donated to DZUP (33.352); 1♀ (LMCI 188-6), with genitalia on slide (GRPM 50-127), emerged on 19.XI.2012, donated to DZUP (33.362).

Additional specimens used for morphological descriptions, with the same collection data as the type material: 1♂ (LMCI 188-5), emerged on 18.XI.2012, mounted on three slides in Canada balsam, genitalia (GRPM 50-124), head and thorax (GRPM 50-125) and wings (GRPM 50-126); three pupae (LMCI 188-8), three last instar larvae (LMCI 188-11), and several galls, dissected from galls induced on *S. weinmannifolius* plants, fixed in Dietrich’ fluid and preserved in 70% ethanol; two last instar larvae, mounted similarly on slides (GRPM 50-128 and 129).

##### Etymology.

The epithet refers to Pampa, a biogeographic province within the Chacoan subregion (*sensu* Morrone 2006), predominantly composed of grasslands, and where *C. pampeanus* was first found.

##### Description of immature stages.

**Figure 5.**
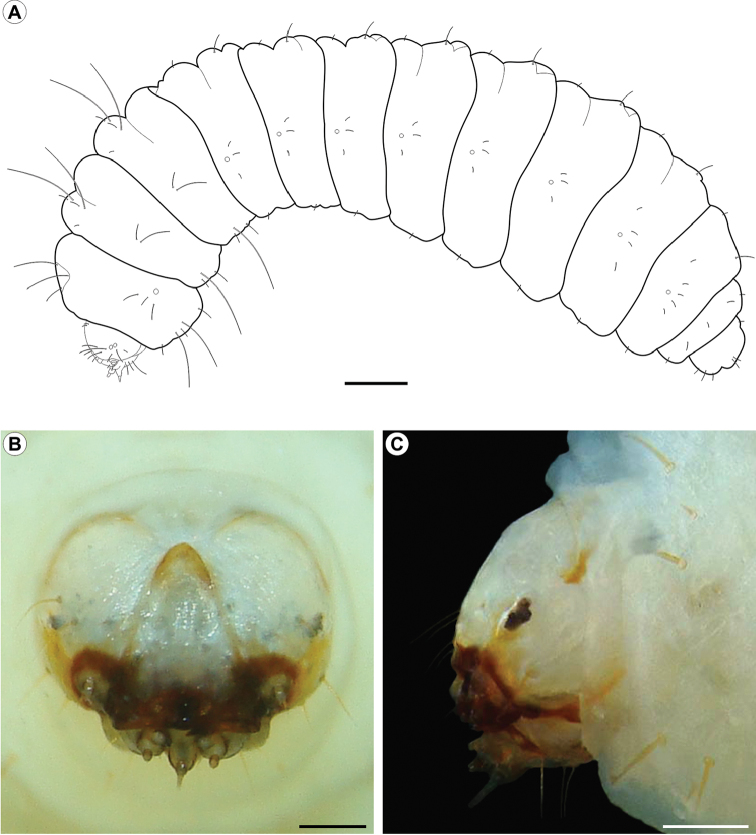
*Cecidonius pampeanus* last larval instar under light microscopy. **A** general schematic representation, lateral view **B, C** head, anterior, and lateral, respectively. Scale bars: 0.5 mm (**A**); 0.2 mm (**B, C**).

**Figure 6.**
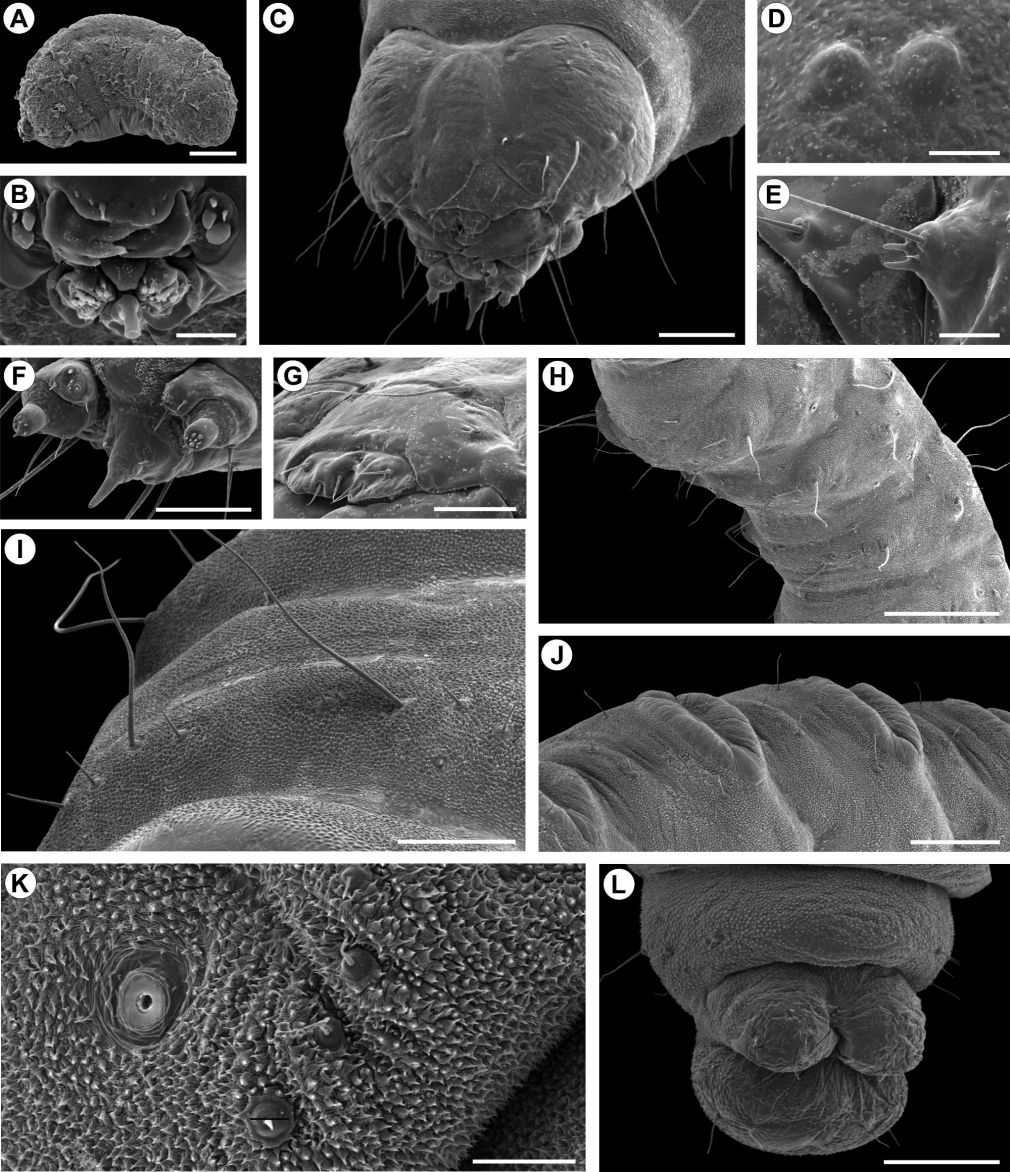
Morphology of *C. pampeanus* first (A, B) and last (C-L) larval instars under scanning electron microscopy. **A** general aspect, lateral view; B, buccal apparatus, anterior **C**, head, latero-dorsal **D** stemmata, lateral; **E**, left antenna, lateral **F** maxilla and labium, antero-lateral **G** labrum and clypeus, latero-dorsal **H** thorax, latero-ventral **I** meso- and metathorax in detail, with aligned setae of different lengths, antero-dorsal **J** second and third abdominal segments, showing tergal calli, latero-dorsal **K** eight abdominal segment in detail, showing aligned secondary setae (arrows) and spiracle, lateral **L** last abdominal segments, ventral. Scale bars: 100 µm (**A, F, G**); 10 µm (**B**); 200 µm (**C**); 50 µm (**D, E, K**); 250 µm (**J**); 1 mm (**H, L**); 0.5 mm (**I**).

*Larva* (Figs 5, 6, 9D, F): With five larval instars, which can be separated from each other by the head capsule width.

*First instar* (Fig. 6A, B). Head capsule width (average + standard error) = 0.066+0.009 mm; body length = 0.570+0.058 mm, n = 4. Head yellowish brown, with chewing mouthparts. Stemmata absent; antennae reduced, located close to mandibles; labrum subquadrate, with three pairs of minute setae; mandibles well developed, with four cusps along distal margin; maxilla with palpus and galea poorly developed; spinneret well developed, tubular; labial palpus one-segmented, bearing an apical sensillum. Thorax and abdomen creamy-white, cylindrical and U-shaped, with no developed primary setae; prothoracic shield, thoracic legs, prolegs, and abdominal calli absent.

*Second instar* (Fig. 9D). Similar in shape and color to fifth instar; head capsule width = 0.160+0.004 mm; body length = 1.060 + 0.134 mm, n = 3.

*Third instar*. Similar in shape and color to fifth instar; head capsule width = 0.217+0.005 mm; body length = 2.078 + 0.052 mm, n = 3.

*Fourth instar*. Similar in shape and color to fifth instar; head capsule width = 0.452+0.017 mm; body length = 3.990 + 0.700 mm, n = 4.

*Fifth instar* (Figs 5, 6C–L, 9F). Head capsule width = 0.898+0.031 mm; body length = 7.190 + 1.722 mm, n = 5. Head yellowish brown, with anterior margin orange-brown and lateral margin convex; frontoclypeus subtriangular, well-marked by pigmented adfrontal sutures, extending to apex of epicranial notch. Two well-developed, latero-located stemmata; antennae 2-segmented, with five sensilla, four short and one ~5x longer the others; labrum slightly bilobed, with three pairs of setae on distal margin; mandible well developed with four cusps along distal margin and one seta basally on external surface; maxilla with palpus and galea reduced; spinneret tubular to conical; labial palpus one-segmented, with well-developed apical seta. Chaetotaxy consisting of 14 pairs of setae: F group unisetose; C group unisetose; A group trisetose; AF group unisetose; P group bisetose, reduced in length; S group trisetose, one reduced in length; SS group trisetose.

Thorax (T) and abdomen (A) creamy-white, cylindrical, slightly curved, covered with microtrichia. Prothoracic shield light yellowish; thoracic legs and abdominal prolegs absent; abdominal segments A2 to A7 with well-developed calli, located on posterior margin of terga. A10 composed of three lobes, one dorsal and two latero-ventral. Circular spiracles without elevated peritreme, laterally on T1, A1–8. Thoracic segments surrounded by short setae interspersed with long ones (~5x longer). T1 with 12 pairs of setae: D group bisetose; XD unisetose; SD unisetose, outside prothoracic shield; L group trisetose, anterior to spiracle; SV group trisetose; MV unisetose; V unisetose. T2-3 with 10 pairs of setae: D group bisetose; SD bisetose; MSD unisetose; L group bisetose; SV group bisetose; V unisetose.

Abdominal segments (AB) with only short setae that are more or less aligned on the middle region of each segment, which are tentatively named. AB1-7 with 6 pairs of setae: D group bisetose; L group trisetose, posterior to spiracles; V unisetose. AB8 with 8 pairs of setae: D group bisetose; SD group unisetose; L group tetrasetose, posterior to spiracles; V unisetose. AB9 with 5 pairs of setae: D group unisetose; SD group unisetose; L group unisetose; SV unisetose; V unisetose. A10 with six pairs of setae: D group bisetose; SD group unisetose; SV trisetose.

**Figure 7.**
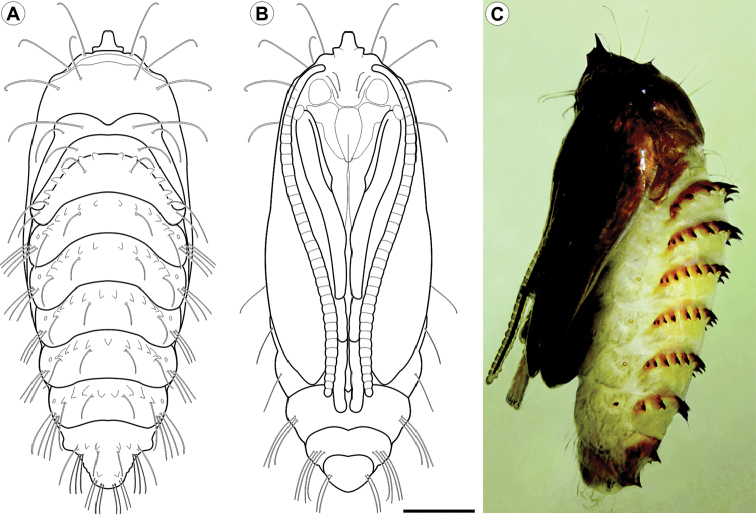
*Cecidonius pampeanus* pupa with light microscopy, under dorsal (**A**), ventral (**B**) and lateral (**C**) views. Scale bar: 1 mm.

**Figure 8.**
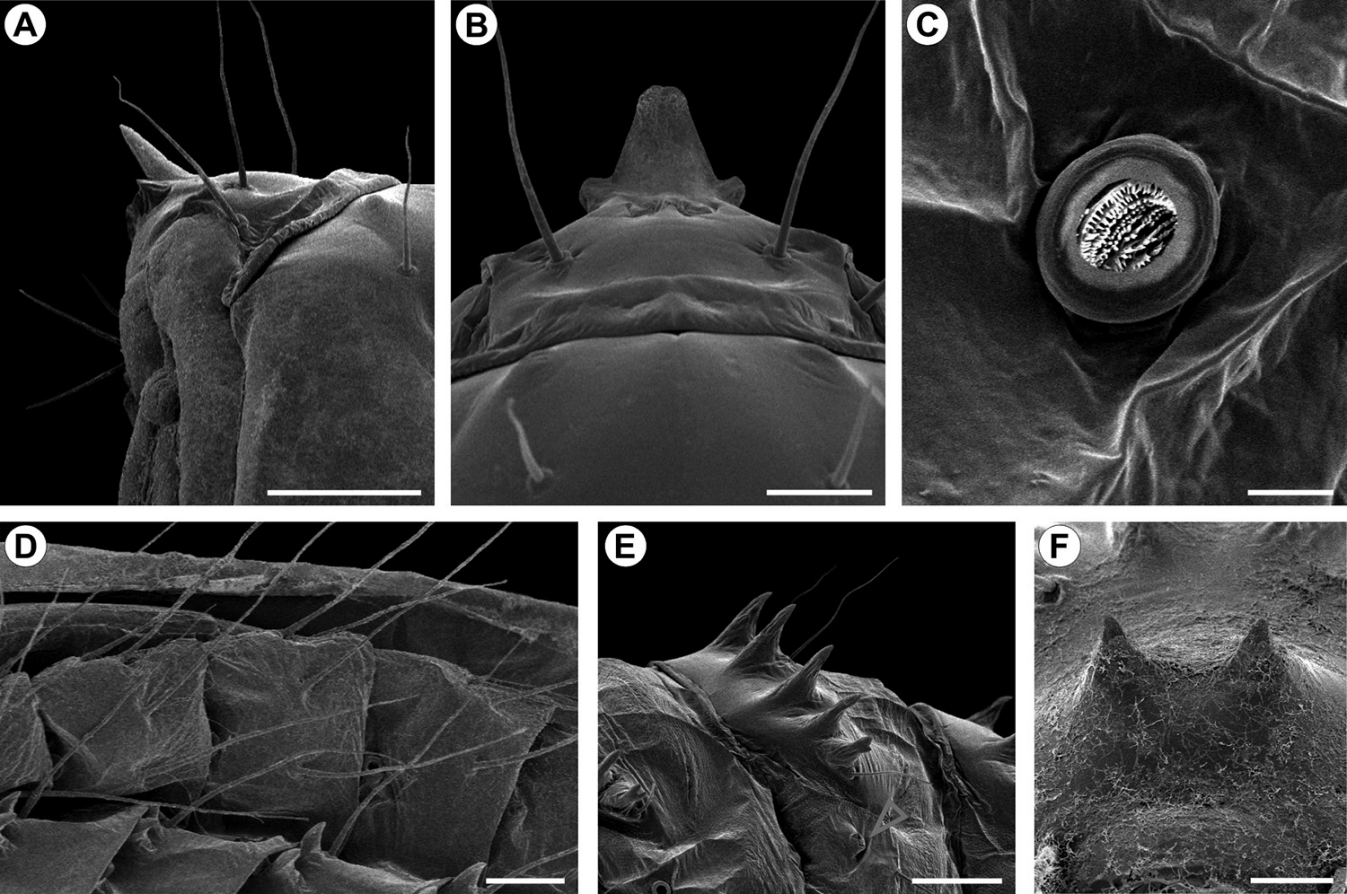
*Cecidonius pampeanus* pupal morphology with scanning electron microscopy. **A, B** head and prothorax, lateral and dorsal views, respectively **C** spiracle of sixth abdominal segment, dorsal **D** subspiracular setae from fourth to sixth abdominal segments, dorsal **E** tergal spines of eighth abdominal segment, lateral (arrow points to partially closed spiracle) **F** spines of tenth abdominal segment, posterior-dorsal. Scale bars: 0.5 mm(**A**); 0.25 mm (**B, C, E**); 0.1 mm (**D, F**).

*Pupa* (Figs 7, 8). Length = 6.44+0.52 mm; n = 3. Yellowish brown, with head, thorax, and abdominal spines becoming dark brown near adult emergence (Fig. 7C). Head with frontal process (gall-cutter) formed by three processes; one large, inverted U-shaped, located in the centre, which is flanked at the base by the other two that are ~5x shorter than the central one, directed laterally and slightly bent to the anterior side (Figs 7, 8A, B). Antennae narrow, long, slightly surpassing forewing apex; prothorax a narrow transverse band between head and mesothorax; hindwings concealed by forewings, reaching posterior margin of sternum A6; pro- and mesothoracic legs extended to A4 and A5, respectively; metathoracic legs reaching beyond forewing apex on segment A7 (Fig. 7). Frons and lateral portion of vertex with two pairs of setae each; tergum T2 with a pair of latero-dorsal setae; tergum T3 with a single seta on each side. Abdominal segments with central region covered by microtrichia; A2–9 with a transverse band of stout spines (Fig. 8E), near anterior margin of terga; tergum A10 with a pair of acute processes on posterior margin (Fig. 8F). Abdominal setae slightly shorter than thoracic, arranged in three rows (dorsal, supra- and subspiracular); one dorsal pair on segments A1–8; one supra-spiracular pair on segments A2–8; four subspiracular pairs on segments A3–6 (Fig. 8D); seven subspiracular pairs on A7-8; six pairs latero-ventrally on A10; spiracles with slightly elevated peritreme, laterally on A2–8, spiracle on A8 partially closed.

##### Natural history.

**Figure 9.**
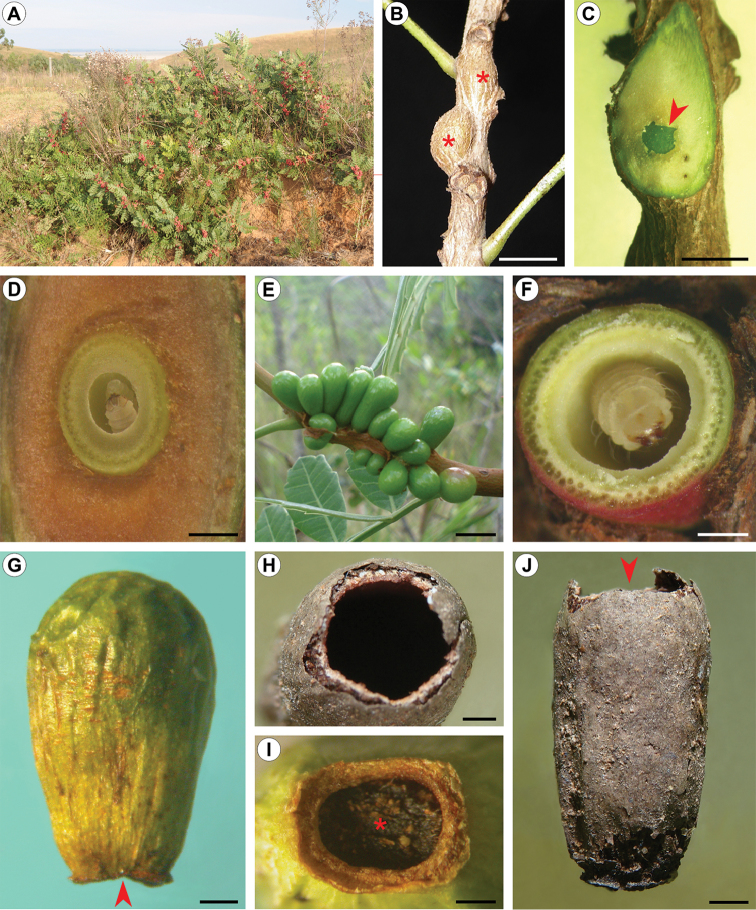
Natural history of *C. pampeanus* on *S. weinmannifolius*. **A** host plant patch at the type locality; **B** young developing galls within swollen stems (asterisks); **C** dissected swollen stem showing developing gall inside (indicated by arrow); **D** transversally sectioned young gall showing second instar larva inside; **E** group of external developing galls on branch; **F** transversally sectioned, full grown gall, showing last instar larva inside; **G** young dehiscent gall; **H** detail of emergence orifice left by adult on distal portion of old, empty gall (pointed by arrow in **J**); **I** detail of young dehiscent gall (arrow in **G**), showing orifice clogged by larval feces (asterisk); **J** old, empty, overwintered gall. Scale bars: 2 mm (**B**); 1 mm (**C, F, G, H, I, J**); 0.5 mm (**D**); 5 mm (**E**).

The unilocular, club-shaped, green galls of *C. pampeanus* develop initially enclosed within swollen stems of *S. weinmannifolius* branches (Fig. 9B, C). Later on in ontogeny, they erupt from the stem surface, either as isolated units or in small groups, and may reach a few tens per branch (Fig. 9F). The larval chamber is almost cylindrical in shape (maximum length = 7.99+0.58 mm; n = 6), and transverse to the stem axis. The external wall is shallow and thinner distally, formed as an expansion of the wood tissue under the bark (Fig. 9D–F). During the last larval instar, *C. pampeanus* galls have their wall somewhat annealed and ruptured at the base (Fig. 9G), when they fall freely to the ground containing the larva inside. The basal orifice left on these galls consequently is clogged by feces (Fig. 9I). These are continually deposited, then dry and solidify at the bottom of the gall chamber. After falling, the gall progressively dries up, turning a dark brown color (Fig. 9J). The external part may appear rotted in some old galls, when thin, longitudinally aligned groves are found on the gall surface. Like *O. argentinana* galls (Moreira et al. 2012), those of *C. pampeanus* also lack an operculum. With the action of the frontal process and body contortions, the pupa opens an irregular orifice on the distal, weaker wall (Fig. 9H). By continuing these movements and anchoring the body laterally with its abdominal spines, the pupa pushes itself partially out of the gall. During this process, the anterior portion of the exuviae is split, allowing adult emergence. In all cases of adult emergence followed under laboratory conditions, the anterior part of the pupal exuviae (head and thorax) was found protruding to the outside, while the posterior third remained in the chamber.

Field collections carried out during five consecutive years at the type locality indicated that *C. pampeanus* is a univoltine species, larvae growing during the summer when young galls are seen on *S. weinmannifolius* stems. Fully developed galls containing last instar larvae have been collected mainly during autumn. Based on several dissections of galls on the ground that were field collected during the winter, it can be inferred that the species overwinters in the larval stage, pupation occurring in spring, and adults emerging later on. This time of the year coincides with full vegetative activity of *S. weinmannifolius* host plants, including production of new sprouts. In the populations of *S. weinmannifolius* located in the study area, several plants can be attacked by *C. pampeanus*, and many branches within a patch of plants can bear galls induced by them. Under severe attack by *C. pampeanus*, *S. weinmannifolius* stems may wilt, die, and then fall, but the underground portion may stay alive. Under low gall densities, however, the aerial portion of plants stay green throughout the year, the signs of detached galls appearing as small, cylindrical craters on their stem surface.

**Figure 10.**
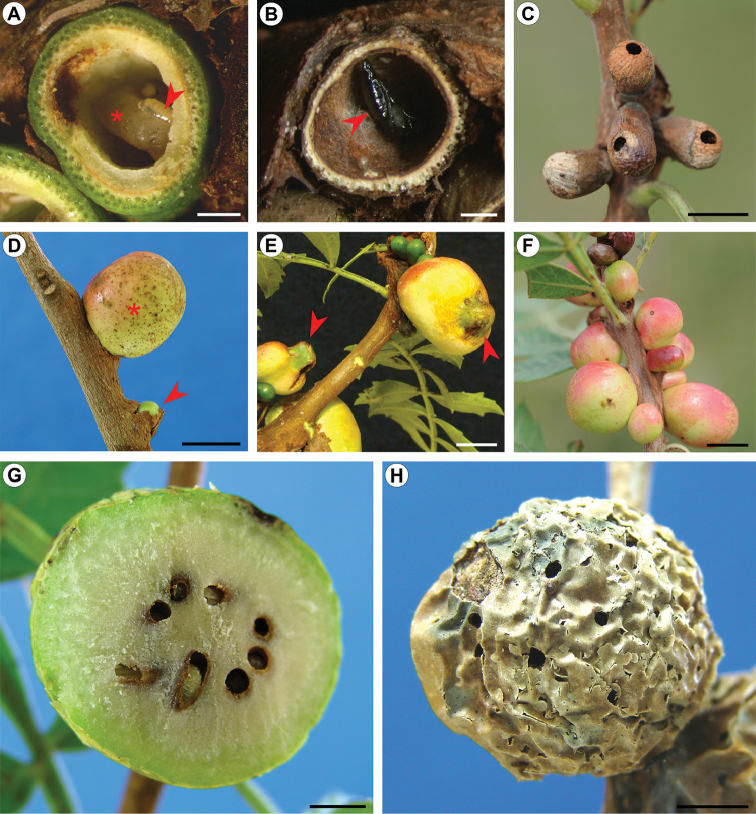
Hymenoptera fauna associated with *C. pampeanus* galls. **A** transversally sectioned, externally developing gall, showing inside a larva of *C. pampeanus* (asterisk) with attached larva (arrow) of *Lyrcus* sp. (Pteromalidae) **B** transversally sectioned, dried gall, with pupa of *Lyrcus* (arrow), after consumption of *C. pampeanus* larva **C** dried and empty attached galls showing orifices of emergence left by adults of *Lyrcus* **D** young, erupting, free of inquiline and adjacent inquiline attacked (*Allorhogas* sp., Braconidae) galls, indicated respectively by open arrow and asterisk **E** young galls of *C. pampeanus* (arrows) partially involved with gall tissue induced by inquilines **F** variation in size among *Allorhogas* galls early attacked **G** a full-developed inquiline-attacked gall showing larvae and pupae in cameras inside **H** senescent *Allorhogas* gall showing orifices of emergence (arrows) left by adults. Scale bars: 1 mm (**A, B, D, F**); 5 mm (**C**); 0.5 mm (**E, G, H**).

In the populations studied here, *C. pampeanus* larvae are only common to find in yearly stages, within those galls still under the bark. Free-living larvae are rarely found in the external galls. These are severely attacked by unidentified parasitoids belonging either to *Lyrcus* Walker (Pteromalidae) or to *Allorhogas* Gahan (Braconidae), whose taxonomy and biology will be treated in detail elsewhere. Larvae of *Lyrcus* are ectoparasitoids found singly attached to *C. pampeanus* larvae inside the galls (Fig. 10A). They suck the internal contains of larvae, killing them and leaving only their exoskeletons intact. These parasitoids pupate inside *C. pampeanus* galls (Fig. 10B), which do not have their main shape changed, but turn a dark brown colour. In this case, galls stay attached to the stems for a longer time compared to ones free of parasitoids. After emergence, adults of *Lyrcus* open a characteristic, small orifice on the distal portion of the gall (Fig. 10C), through which they leave. By contrast, larvae of *Allorhogas* are gregarious and inquilines. They modify *C. pampeanus* galls, inducing production of additional tissue. When initially viewed externally in this case, *C. pampeanus* galls appear partially surrounded by this type of tissue (Fig. 10E). Later in ontogeny they are completely involved by such tissues, turning into globular, pinkish, large galls (up to 3.2 cm in diameter; n = 8) that last much longer in the field and promptly call attention (Fig. 10D, F). These galls are multilocular; larvae of inquilines are found individually in several chambers within (Fig. 10G). Pupation also occurs inside galls, that then dry up and turn dark brown; adults of inquilines leave through small circular orifices that are found on the gall surface (Fig. 10H).

##### Host-plant and distribution.

**Figure 11.**
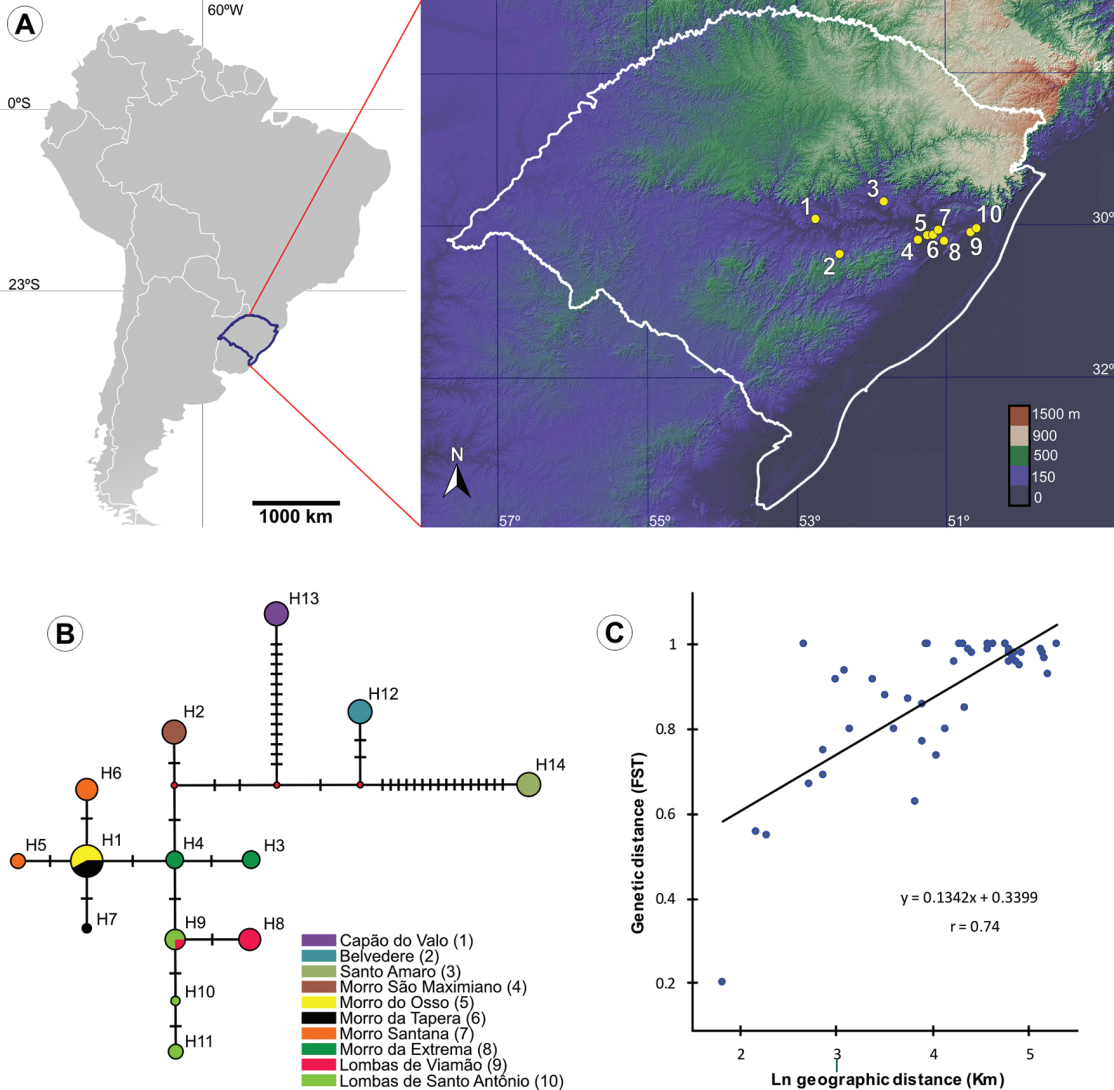
Geographic distribution and genetic variation among populations of *C. pampeanus* within Rio Grande do Sul State, Brazil; **A** localities of populations studied (see Suppl. material 3 for exact geographic coordinates and elevations) **B** evolutionary relationships of COI haplotypes across ten populations. The circles represent haplotypes; the diameter is proportional to the frequency in 60 analyzed individuals. Small red circles indicate intermediate vectors. Transversal bars represent mutational steps. Numbers in parentheses correspond to localities in the map (**A**) **C** correlation between pairwise geographic distance and estimates of gene flow (φST) (P<0.05).

Galls of *C. pampeanus* have been found only on branches of *Schinus weinmannifolius* Mart. ex Engl. (Anacardiaceae). This is a small shrub (up to 50-cm tall), originally found scattered in open savannas (Fig. 9A), hill tops and forest borders of southern South America, including central and south Brazil, Paraguay, northeast Argentina and Uruguay (Barkley 1957, Luz 2011). However, populations of *S. weinmannifolius* bearing galls of *C. pampeanus* were found only in Rio Grande do Sul, the southernmost state of Brazil, particularly in the surroundings of Porto Alegre city (Fig. 11A) in the eastern limit of the Pampean province within the Chaco biogeographic region (*sensu* Morrone 2006). This region, also known as the Southeastern Highlands, since it reaches higher elevations than the remaining Pampean areas, includes several low-elevation hills (up to 300 m) that are more or less interwoven with fragments of semi-deciduous forests, herbaceous and shrub vegetation and single-layer grasslands, forming a mosaic. In this area a few, isolated, populations of *S. weinmannifolius* were found either as isolated plants or forming small patches (up to 3m in diameter), primarily located on hilltops and hill slopes, and a few scattered in the single-layer grasslands that prevail in the lower elevation areas.

Little is known about the biology or natural history of *S. weinmannifolius*. Although also found as isolated individuals, it usually forms small patches of plants, particularly in sandy soils. Preliminary field observations suggest that *S. weinmannifolius* is perennial, having a subterraneous habit of growth, forming stolons that grow just below ground and from which new sprouts emerge every year, starting in spring. At the type locality, the first flowers appear during November and the flowering season may last until March; fruits are found on plants from December to May. There is apparently little if any vegetative growth during the winter, which is also the season when the aerial parts of *S. weinmannifolius* plants may wilt and die.

##### Population genetic structure.

Inferences on the genetic variability of *C. pampeanus* resulted from 42 (3%) variable sites. Overall, haplotype (Hd) and nucleotide diversity (π) were 0.92±0.01 and 0.0007±0.0009, respectively (Table 2). Individual populations presented Hd from 0.33 to 0.73 (P9 and P10, respectively) and π from 0.002 to 0.0013 (P9 and P10, respectively). A total of 14 haplotypes were found in ten populations (Table 2; Fig. 11B). We found only one haplotype in each in P1 to P5; therefore, standard diversity indices and neutrality tests were not performed. From P6 to P9 two haplotypes per site were observed; P10 presented three haplotypes, the highest diversity. Except for H1 and H9, which were shared between P5/P6 and P9/P10 respectively, all were unique to each locality (Fig. 11B). Characterization of pairwise gene flow based on the F_ST_ index indicated significantly high levels of genetic structure in populations of *C. pampeanus*. Overall, F_ST_ ranged from 0.55 to 1 (P < 0.05) (Table 3). The lowest level observed was 0.20, between P5 and P6, not significant (P > 0.05). Spatial genetic structure assessed by the correlation between genetic and geographic distances indicated a significant pattern of isolation by distance for the ten populations (r = 0.74, P < 0.01) (Fig. 11C). Quantitative differentiation based on two groups of comparison reinforced the structure by distance pattern (Suppl. material 2). Both analyses (Jacuí River as a barrier and geographic distance) found similar values of F_ST_ (0.97; P < 0.001). However, when we grouped P2 with the cluster formed by P4 to P10 the divergence among groups was lower (46.45%; P < 0.001) than when we grouped it with P1 and P3 (58.73%; P < 0.001). Similarly, the divergence among populations within groups decreased from the first to the second scenario (51%, P < 0.01; 39.15%, P < 0.001, respectively).

**Table 3.** Pairwise estimates of gene flow based on φ-statistics (φ_ST_) for cytochrome oxidase subunit I mitochondrial sequences in nine populations *C. pampeanus*. All comparisons were statistically significant (P < 0.05), except the value in bold.

	Capão do Valo	Belvedere	Santo Amaro	Morro São Maximiano	Morro do Osso	Morro da Tapera	Morro Santana	Morro da Extrema	Lombas de Viamão	Lombas de SantoAntônio
Capão do Valo	–									
Belvedere	1.0000	–								
Santo Amaro	1.0000	1.0000	–							
Morro São Maximiano	1.0000	1.0000	1.0000	–						
Morro do Osso	1.0000	1.0000	1.0000	1.0000	–					
Morro da Tapera	0.9846	0.9636	0.9875	0.9200	**0.2000**	–				
MorroSantana	0.9814	0.9583	0.9848	0.9166	0.6666	0.5500	–			
Morro da Extrema	0.9818	0.9538	0.9853	0.8800	0.8000	0.6909	0.7466	–		
Lombas de Viamão	0.9906	0.9787	0.9923	0.9565	0.9411	0.8631	0.8695	0.8000	–	
Lombas de Santo Antônio	0.9682	0.9276	0.9740	0.8521	0.8000	0.7368	0.7652	0.6285	0.5600	–

Finally, analysis of demographic history by mismatch distribution indicated an overall multimodal pattern for *C. pampeanus* that is not compatible with a scenario of recent demographic expansion (Suppl. material 4). Single population analysis indicated a unimodal pattern, particularly for P9 that showed a possible scenario of expansion. In addition, overall neutrality tests yielded positive and non-significant values for all indices with respect to neutral expectations (Table 2). Single populations presented positive values, except P7 that showed negative values (but non-significant) for some parameters (i.e., Tajima´s D and Fu and Li’s D and F) and P9, that presented all negative (but non-significant) values.

**Table 2.** Summary of genetic variability of ten populations of C. pampeanus based on mitochondrial DNA sequences. Populations (Pop) are as follows: Capão do Valo (CV), Belvedere (Bel.), Santo Amaro (SA), Morro São Maximiliano (MSM), Morro do Osso (MO), Morro da Tapera (MT), Morro Santana (MS), Morro da Extrema (ME), Lombas de Viamão (LV) and Lombas de Sto. Antonio (LSA). Numbers from 1 to 14 indicate number of haplotypes found in each population. Hd, haplotype diversity; π, nucleotide diversity. Neutrality tests performed: Tajima´s (D); Fu and Li’s (D and F); Fu's (Fs). Asterisks indicate significant values, P < 0.05.

Pop.	Haplotypes															π	Neutrality tests			
																	Tajima’s	Fu and Li’s		Fu´s
	1	2	3	4	5	6	7	8	9	10	11	12	13	14	Hd		D	D	F	Fs
CV													X		0.00	0.0000	–	–	–	–
Bel.												X			0.00	0.0000	–	–	–	–
SA														X	0.00	0.0000	–	–	–	–
MSM		X													0.00	0.0000	–	–	–	–
MO	X														0.00	0.0000	–	–	–	–
MT	X				X										0.53	0.0004	0.850	1.052	1.029	0.625
MS						X	X								0.33	0.0005	-1.131	-1.155*	-1.195	0.952
ME			X	X											0.60	0.0008	1.753	1.279	1.434	1.938
LV								X	X						0.33	0.0002	-0.933	-0.950*	-0.964	-0.003
LSA									X	X	X				0.73	0.0013	1.647	1.395	1.523	0.758
Overall															0.92	0.0068	-0.1362	1.775*	1.2683	2.886

## Discussion

### Taxonomy and phylogeny

Since it was proposed as a family by Brèthes (1916), the position of Cecidosidae remained for a long time uncertain until its affinity to the superfamily Adeloidea was clarified by Becker (1977), who regarded the group as endemic to South America. The affinity of *Scyrotis* with South American cecidosids was proposed later by Davis (1987). Molecular data provided here give further support to this taxonomic affinity, and show that the African *Scyrotis* are much older (*ca.* 90 Myr) than South American genera. Results also suggest there could exist more than one cecidosid lineage in Africa, since the two species we sequenced were 27% apart from each other in terms of genetic divergence in our analyses. The first studies on African cecidosids conducted by Meyrick (1909, 1913, 1928) clearly suggested the existence of at least three lineages of cecidosids, which can be separated by differences in the buccal apparatus of the adults. In fact, these lineages were initially grouped by him into different genera, which were later considered by Gozmány and Vári (1973) as synonyms of *Scyrotis* and treated as such ever since (for a detailed discussion, see Mey 2007). Two of these lineages are represented in our analysis by *Scyrotis* sp. and *S. granosa*; the former presents a rudimentary proboscis and maxillary palpi and the latter lacks such buccal structures. This question should be taken into account in revising the taxonomy and phylogeny of the family in the future, which is much needed. Although not linked to any *Scyrotis* species in particular, a field survey of galls conducted by van Noort et al. (2007) found them in association with several species of *Searsia* and suggested the existence not only of a wide variety of gall morphology but also considerable variation in life history styles among the African *Scyrotis*. It is unlikely that such variation will be conciliated within a single genus, which should be further explored. Unfortunately, this revision is pending upon description of the immature stages and gall morph types they induce, but as already said these aspects are still unknown for any African cecidosid species. The present study showed how valuable the inclusion of immature stages and galls is in taxonomic studies of cecidosids, whose adults in particular have relatively uniform morphology, especially regarding the genitalia (Mey 2007). In addition, our results support an accelerate evolutionary rate in all Cecidosidae lineages, as mentioned by Pellmyr and Leebens-Mack (1999) when a cecidosid (*C. eremita*) was used for the first time in a molecular phylogeny of Adeloidea. Similarly, Regier et al. (2015) in a family-level phylogenetic study based on 19 genes, found a high substitution rate in Cecidosidae when including *C. eremita* and *D. capsulifex*, which they found in Incurvariidae as well. This faster evolution of cecidosids makes it difficult to resolve some internal evolutionary relationships within the group, generating phylogenetic uncertainties that are hard to overcome even by increasing markers, and should be further explored.

*Cecidonius* gen. n. resulted as a unique lineage in the present study from both morphological and molecular analyses. Also, interestingly, it appeared as one of the most recent lineages (*ca.* 24 Myr) to be evolved within the extant cecidosids. It diverged *ca.* 16% from the closest related lineage, an additional undescribed cecidosid taxon existing in Chile and Argentina, which was included in the present study for comparison. This undescribed taxon differs from *Cecidonius* by having adults that lack a rudimentary proboscis and having a three-segmented maxillary palpus, pupae bearing a gall-cutter with a different shape, larvae without long hair on thorax and galls with completely developed wall without basal orifice, and will be described elsewhere. Molecular findings also showed that although described as monotypic, there is at least one more species belonging to *Cecidonius*, associated with *Schinus terebinthifolius* Raddi, which is still awaiting description. This undescribed species diverged from *C. pampeanus* by more than 9% in DNA sequences. Its galls are conspicuous, morphologically different, and larger than those of *C. pampeanus*. They are relatively common in populations of *S. terebinthifolius* existing in southern Brazil. Unfortunately, we have no pupae or adults of this species yet, which apparently shares a similar life-history style and associated difficulties regarding rearing of *C. pampeanus*.

### Life history

It took us a few years to obtain the small number of *C. pampeanus* pupae and adults used for description in this study. Although relatively abundant as young larvae when still under the bark of swollen stems, later instars of *C. pampeanus* occur at low density in the field. Collection of mature, dehiscent galls during later summer, either using cloth bags attached to the plants or picking by hand those that had naturally dropped to soil, always led to failure regarding development under laboratory conditions. Dissection of these galls demonstrated that larvae do not pupate, remaining alive in the last larval instar for months, eventually dying without any apparent cause. Interestingly, similar difficulties regarding rearing of *C. pampeanus* are also mentioned by Meyrick (1917) in relation to the African *Scyrotis*. *Cecidonius pampeanus* apparently diapauses for months in the last instar larva, which stays motionless within its dehiscent gall in the soil until pupation and adult emergence occur in the next growing season. Probably this species presents a seasonal adaptation (*sensu* Tauber, Tauber and Masaki 1986) to overcome the unfavourable low temperatures that prevent growth during winter, and also adjust its life cycle to the host plant phenology. As already mentioned, new growth shoots that are required for gall induction (Raman 1994, Yukawa 2000) start appearing on *S. weinmannifolius* plants during the spring. This time of the year coincides with adult emergence in the field, and supposedly also with oviposition in *C. pampeanus*. A group of approximately 20 galls were collected in the field by the first author during winter and kept under room temperature in the laboratory within plastic vials containing moist soil from the type locality. A few were dissected at fifteen-day intervals, rendering only last instar larvae. The first pupa in this case appeared in spring (October), and the adults, which were used in the present description, *ca.* one month later. The token stimuli that initially trigger and later break the diapause in *C. pampeanus* remain to be determined. We may speculate from above that the corresponding stimuli may be received during autumn by the dehiscent galls that are already in the soil.

### Inquiline and parasitoid wasps

*Allorhogas* species are among a few braconid wasps having a phytophagous feeding habit. They are apparently relatively common and widespread in the Neotropics, all associated with galls, occurring in several plant families including Burseraceae, Fabaceae, Melastomataceae, Polygonaceae, Rubiaceae, and Solanaceae (e.g., Macedo and Monteiro 1989, Marsh et al. 2000, Marsh 2002, Penteado-Dias and Carvalho 2008, Chavarría et al. 2009, Centrella and Shaw 2010, 2013, Martínez et al. 2011, Martínez and Zaldívar-Riverón 2013). However, their biology is largely unknown, and it is still uncertain whether they are primary gall inducers or inquilines. A clear pattern always emerged during dissections of hundreds of galls from several *S. weinmannifolius* populations in the present study, demonstrating that they are inquilines. First, they were never found inside young galls that were located under swollen stem bark, where only young larvae of *C. pampeanus* were always present. Second, erupted galls bearing either free-living *C. pampeanus* larvae or those attacked by *Lyrcus* ectoparasitoids do not change their shape, but only those bearing *Allorhogas* that turn from cylindrical into globular galls. Third, *Allorhogas* immatures were always found within older, erupted and much larger, shaped-modified galls, where larvae of *C. pampeanus* were found dead. Fourth, as already described, a progressive transition in shape between galls free from such inquilines (cylindrical) to those attacked by them (globular) is found in the field, always in association with early development of *Allorhogas* larvae. Most of the *Allorhogas* studies listed above have a taxonomic bias and are based on descriptions of adults reared during extensive surveys, without including descriptions of immature stages. They lack information on gall ontogeny, and most importantly, about identification of trophic levels of insects present within these galls. Thus, the biological status of *Allorhogas* in those gall systems should be re-examined, since some of them may not induce galls but act as inquilines, the true gall inducers being either underexplored or missed in such cases.

Similar to what was described for the *Scyrotis* galls attacked by *Rhoophilus* Ioewi (Hymenoptera: Cynipidae) inquilines (van Noort et al. 2007), space for a *C. pampeanus* larva within a given gall is progressively diminished with the development of tissues induced by *Allorhogas* larvae. In fact, in several cases in the present study during the dissections of medium-sized developing galls bearing *Allorhogas* inquilines, a dead *C. pampeanus* larva was found within a compressed space inside. From a gross morphology perspective, tissues induced to develop by *Allorhogas* are clearly different from those induced by the original inducer *C. pampeanus*, regarding thickness, consistency, and colour. In general, tissues present in insect galls are complex, and may structurally vary even within a given gall lineage (Stone and Schönrogge 2003). Specially the nutritious ones, which are absent on ungalled host plants, may also vary in complexity at a very fine scale not only among but also within galls. For example, when tissues produced by lepidopterans and hymenopterans are compared, differences between them emerge at the cell level in relation to the type, quantity, local and disposition of chemicals they store, among other characteristics (e.g., Ferreira and Isaias 2013, Vecchi et al. 2013). These tissues are used for feeding by the corresponding inducers independently, that is within their own distinctly located galls. This is not the case in the present system, where such tissues are induced by distantly related insect lineages and occur within the same gall. Thus, we suggest that tissues induced by *Allorhogas* species may inhibit feeding by *C. pampeanus*, whose larvae, by being confined in space, completely surrounded by tissues unsuitable for feeding, would be lead to death by inanition.

Additional field observations suggest that the existence of an inquiline association between *Allorhogas* species and galls of other cecidosids is common in southern Brazil. This is the case of the gall induced in *S. terebinthifolius* by the undescribed, additional species of *Cecidonius* already mentioned, as well as of those induced in *S. polygamus* by *C. eremita* and *E. minutanus*. Thus it seems that these braconid wasps parallel in the Neotropics the cynipd wasps that are inquilines of cecidosid galls in Africa (van Noort et al. 2007). Cynipids are found in South America, not acting as inquilines but as primary gall inducers, as for example in Fabaceae (e.g., Nieves-Aldrey and San Blas 2015). Unfortunately, little is known about the biology of the Neotropical species of *Lyrcus*. They are diverse and difficult to identify in the Nearctic region, where many are important parasitoids of agricultural pests, primarily belonging to Coleoptera and Diptera (Gibson GAP, Agriculture and Agri-Food Canada, pers. comm.). According to preliminary observations there are additional insect species yet to be explored in association with *C. pampeanus* galls, including cecidophages, predators, hyperparasitoids and successors that use them as shelter. The latter may include other arthropods and are common in cecidosid galls, since some of these galls may last for years after adult emergence and thus be used by other insects for shelter and even for nesting (e.g., Wille 1926, Laroca 1972). We hope this study will stimulate additional studies on this topic, thus revealing fully the hidden diversity existing in association with these galls.

### Genetic diversity and conservation biology

Our study provides strong evidence that *C. pampeanus* is under threat of extinction, and protection measures should be taken to conserve its remaining populations. The reasons are based primarily on the destruction of the host plant habitat. Open savannas of southern Brazil (= Brazilian ‘Campos’) where populations of *S. weinmannifolius* are found have been suffering from anthropic influence for decades, mostly caused by agriculture in general and/or cattle ranching, and recently from widespread expansion of *Eucalyptus* L’Heritier, *Acacia* Martius and *Pinus* Linnaeus plantations (Overbeck et al. 2007, Cordeiro and Hasenack 2009). A search by the first author for populations of *S. weinmannifolius* of which older dried material is preserved in the main herbaria in the region (e.g., UFPR/ Curitiba, Barbosa Rodrigues/Itajai; and UFRGS/Porto Alegre), suggested that most of these have disappeared since. In Parana state, for example, extant populations seem to be restricted to a few places, including the preserved area of Parque de Vila Velha, Ponta Grossa municipality. In Rio Grande do Sul scattered populations were located on high elevation steppes, as for example in Canela and São Francisco de Paula municipalities, and particularly at low elevations in the western portions of the Pampa biome. However, as already mentioned, extant populations of *S. weinmannifolius* bearing galls of *C. pampeanus* were restricted to small patches in the latter area. More importantly, these populations are distant and isolated from each other. Most of them are located at higher elevation, such as on hilltops and hill slopes interspersed with small bushes as already mentioned, where they are relatively more protected from anthropic influence. At least two of these areas (Morro do Osso and Morro Santana) are officially protected areas already, but the remaining populations are located on private property. *Schinus weinmannifolius* is considered a pasture weed, supposedly unpalatable to livestock, the reason for which we presume it has disappeared from most low elevation areas, where agriculture and cattle ranching prevail as the main economic activities. *Schinus weinmannifolius* is apparently heliophilous, and in consequence does not grow satisfactorily within plantations such as those composed of *Eucalyptus*, *Acacia* or *Pinus*, also common in the region.

There is no indication that adults of cecidosids feed actively, last long and disperse much; oviposition supposedly occurs on the plants surrounding those where they developed as immatures (e.g., San Blas and Davis 2013). The limited dispersal together with low connected patches of *S. weinmannifolius* resulted in ‘island’ populations of *C. pampeanus* with reduced variability. Moreover, high genetic structure and partition of variation based on geography corroborated a pattern of isolation by distance. The restricted distribution and small population sizes are important causes of reduced genetic diversity (Hamrick and Godt 1989), since the effects of natural selection and/or demographic changes may be more pronounced in such populations (Ellstrand and Elam 1993, Gibson et al. 2008). The low variability found in *C. pampeanus* possibly makes the species vulnerable for novel selection pressure. Whether populations would be affected by stochastic processes, particularly genetic drift, depends on gene flow within and among populations, among other ecological factors. We found significantly low levels of gene flow among populations of *C. pampeanus*. Haplotypes were mainly unique to each locality, except between Morro do Osso/Morro Tapera and Lombas de Viamão/Lombas de Santo Antônio; even so, the latter presented significant high F_ST_ values.

The low number of nucleotide differences between the haplotype pairs (except for H12, H13 and H14) and a multimodal curve in the mismatch distribution analysis of *C. pampeanus* indicate that population expansion is unlikely to have occurred. In contrast, the population of Lombas de Viamão presented an expansion pattern. According to Rogers and Harpending (1992) and Tajima (1989), bottlenecks may generate waves in the distribution of pairwise nucleotide differences. However, contrary to expansion, a population contraction leads to maintenance of genetic diversity over time. In a bottleneck model individuals differ in the average number of nucleotide changes when taken randomly from a given population. Such an effect leads to multipeak nucleotide distributions, as well as large pairwise differences between them (Harpending et al. 1998, Rogers and Harpending 1992). Additionally, when estimated by median-joining, the haplotype topology also did not support a population expansion scenario, as it did not fit into a typical star-like model (Harpending et al. 1998, Slatkin and Hudson 1991). The results suggest that demographic changes in populations of *C. pampeanus* are a consequence of ancient historical processes and recent decline, likely due to landscape disturbance.

The above-mentioned higher trophic level-associated fauna may be also under threat, considering that its existence depends on the success of *C. pampeanus*, the primary gall inducer. In other words, a whole community associated with *C. pampeanus* galls may go extinct in South Brazil, even before species that integrate it have been described, in the case of extinction of the primary gall inducer. A survey should be carried out to identify the unknown fauna associated with these galls. We also suggest that additional studies should examine the degree of specificity and inter-dependence of this fauna with *C. pampeanus* and its host plant. These actions should be prioritized when planning the corresponding conservation measures, since they are prerequisite to their implantation. Protection measures have been scarcely taken in relation to the lepidopteran species that are under threat of extinction in the Neotropical region. In Brazil, actions in this regard have involved primarily the butterflies, in total 55 species that are officially considered under threat of extinction (Freitas and Marini-Filho 2011). However, microlepidoptera and associated plants are largely unknown in this country due to a corresponding taxonomic impediment (Aguiar et al. 2009), and thus they have been completely neglected from a conservation biology perspective. Within the gall-inducer and leaf-miner micromoths there are many species that are specialists on rare and/or endemic plants, particularly in the Brazilian Atlantic Forest, most yet to be discovered and/or described (Luz et al. 2014, Moreira et al. 2017). By being dependent on endemic hosts at a regional scale, these species in particular are under comparatively greater threat, because most of such plants are restricted in distribution (Lewinsohn et al. 2005). This study is apparently the first to suggest that a micromoth and its associated fauna should be taken into account in this regard in Brazil. It is also important to emphasize that the restricted number of extant *C. pampeanus* populations are located within the southern Brazilian “Campos” (= Pampean savanna) that is considered a diverse but neglected biome from a conservation biology perspective (Overbeck et al. 2007), and where no moth has ever been targeted from a conservation biology perspective.

### Further remarks

This study also showed how important intensive, integrative taxonomic studies are to identify accurately the role of a cecidosid species in a given gall community. *Cecidonius pampeanus* attracted our attention *ca.* 10 years ago as a cecosid lineage by comparison of DNA sequences extracted from the larval stage, dissected from under the bark of swollen stems of *S. weinmannifolius*. For several years, its identification remained provisional, tied only to DNA similarity to other cecidosids, since for this new species morphology of the last larval instar, found later in the field, was also atypical compared to any known cecidosid. Full confirmation of the existence of this new lineage came when we finally obtained their pupae and reared them to adults. We inferred that the absence of such an approach led Tavares (1909: 8) to identify the true inducers of such *S. weinmannifolius* galls as “… *probabiliter Cynips incognita*” [… probably an undescribed *Cynips* Linnaeus species]. This action has prevented unraveling not only the true gall inducer, but also the diversity of fauna associated with such galls for more than a century, since his rationale was followed without being questioned by other authors (e.g., Wille 1926, Houard 1933, Sáiz and Núñez 1997). In other words, from Tavares’ original description until the present study, such galls have been treated as two trophic level systems, and their induction was erroneously associated with an unidentified species of Cynipidae (Hymenoptera). The Portuguese Jesuit priest Joaquim da Silva Tavares, also a naturalist who first described these galls, was a pioneer in the study and description of Brazilian cecidology during the first quarter of the last century. His descriptions were accurate and finely illustrated, but most of them were based on the gall morph type only, not always being associated with precise identification of the corresponding inducers. We suppose the large and colourful *Allorhogas*- bearing galls, which appear as neat black and white photographs in his publication (Plate VIIi, figs 22, 23), called his attention to *S. weinmannifolius* plants at first sight in the field. As already mentioned, free-living external galls bearing *C. pampeanus* larvae are rarely found on *S. weinmannifolius* plants in the field, most being killed by *Lyrcus* parasitoids, and thus they may never have been encountered by him. That is, the gall phenotype that is modified by the *Allorhogas* inquilines would have misguided him and led him to suggest the primary induction of such galls to be by cynipid wasps, based on the immature stages obtained when dissecting such galls, since those dissections were also illustrated by him (Plate VIII; figs 24, 25). He apparently did not rear to the adult stage of the assumed cynipid species at that time, since later on when working with the Brazilian melastomatacean galls he made comments on his disappointment about not ever having had a Brazilian cynipid specimen in his collection (Tavares 1917, p.19). In the same publication, he indirectly admitted having erroneously thought at first that these melastomatacean galls also looked like those induced by cynipids in Europe, but that he had changed his mind after having surprisingly obtained the first adult Lepidoptera reared from them.

## Supplementary Material

XML Treatment for Cecidonius

XML Treatment for Cecidonius pampeanus

## Supplementary materials

Supplementary material 1 Table S1. Data type: molecular data Explanation note: Primers and conditions used in polymerase chain reaction (PCR) to amplify COI, 16S and Wg genes. This dataset is made available under the Open Database License (http://opendatacommons.org/licenses/odbl/1.0/). The Open Database License (ODbL) is a license agreement intended to allow users to freely share, modify, and use this Dataset while maintaining this same freedom for others, provided that the original source and author(s) are credited.Gilson R.P. Moreira, Rodrigo P. Eltz, Ramoim B. Pase, Gabriela T. Silva, Sérgio A.L. Bordignon, Wolfram Mey, Gislene L. Gonçalves

Supplementary material 2 Table S2. Data type: statistical data Explanation note: Analysis of Molecular Variance (AMOVA) using φ-statistics based on cytochrome oxidase subunit I mitochondrial sequences for groups of *C. pampeanus*, defined according to different dispersal barriers. This dataset is made available under the Open Database License (http://opendatacommons.org/licenses/odbl/1.0/). The Open Database License (ODbL) is a license agreement intended to allow users to freely share, modify, and use this Dataset while maintaining this same freedom for others, provided that the original source and author(s) are credited.Gilson R.P. Moreira, Rodrigo P. Eltz, Ramoim B. Pase, Gabriela T. Silva, Sérgio A.L. Bordignon, Wolfram Mey, Gislene L. Gonçalves

Supplementary material 3 Table S3. Data type: specimens data Explanation note: Specimens used in this study for phylogenetic reconstruction and genetic structure analysis of *C. pampeanus*. This dataset is made available under the Open Database License (http://opendatacommons.org/licenses/odbl/1.0/). The Open Database License (ODbL) is a license agreement intended to allow users to freely share, modify, and use this Dataset while maintaining this same freedom for others, provided that the original source and author(s) are credited.Gilson R.P. Moreira, Rodrigo P. Eltz, Ramoim B. Pase, Gabriela T. Silva, Sérgio A.L. Bordignon, Wolfram Mey, Gislene L. Gonçalves

Supplementary material 4 Figure S1. Data type: statistical data Explanation note: Graphs depicting the results of the mismatch distribution analysis for the total samples (*C. pampeanus*) and populations alone (P6 to P9). The analysis was performed with 1420 bp of COI sequences (excluding all sites with missing information or gaps). This dataset is made available under the Open Database License (http://opendatacommons.org/licenses/odbl/1.0/). The Open Database License (ODbL) is a license agreement intended to allow users to freely share, modify, and use this Dataset while maintaining this same freedom for others, provided that the original source and author(s) are credited.Gilson R.P. Moreira, Rodrigo P. Eltz, Ramoim B. Pase, Gabriela T. Silva, Sérgio A.L. Bordignon, Wolfram Mey, Gislene L. Gonçalves

Supplementary material 5 Figure S2. Data type: statistical data Explanation note: Neighbor-Joining tree of *Cecidonius pampeanus* with the evolutionary distances computed using the Kimura 2-parameter method based on 1.6 Kb of cytochrome oxidase sequences. The analysis involved 60 individuals from 10 populations. This dataset is made available under the Open Database License (http://opendatacommons.org/licenses/odbl/1.0/). The Open Database License (ODbL) is a license agreement intended to allow users to freely share, modify, and use this Dataset while maintaining this same freedom for others, provided that the original source and author(s) are credited.Gilson R.P. Moreira, Rodrigo P. Eltz, Ramoim B. Pase, Gabriela T. Silva, Sérgio A.L. Bordignon, Wolfram Mey, Gislene L. Gonçalves

## References

[B1] Aguiar AP Santos BF Couri MS Rafael JA Costa C Ide S Duarte M Grazia J Schwertner CF Freitas AVL Azevedo CO (2009) Insecta. In: Rocha RM, Boeger WA (Orgs) Estado da Arte e Perspectivas para a Zoologia no Brasil. Editora UFPR, Curitiba, Brazil, 131–155.

[B2] Bandelt HJ Foster P Röhl A (1999) Median-joining networks for inferring intraspecific phylogenies. Molecular Biology and Evolution 16: 37–48. https://doi.org/10.1093/oxfordjournals.molbev.a026036 10331250 10.1093/oxfordjournals.molbev.a026036

[B3] Barkley FA (1957) A study of *Schinus* L. Lilloa 28: 1–110.

[B4] Becker VO (1977) The taxonomic position of the Cecidosidae Brèthes (Lepidoptera). Polskie Pismo Entomologiczne 47: 79–86.

[B5] Bouckaert R Heled J Kuhnert D Vaughan T Wu C-H Xie D Suchard MA Rambaut A Drummond AJ (2014) BEAST 2: a software platform for Bayesian evolutionary analysis. PLoS Computational Biology 10: e1003537. https://doi.org/10.1371/journal.pcbi.1003537 10.1371/journal.pcbi.1003537 PMC3985171 24722319

[B6] Brèthes J (1916) Estudio fito-zoológico sobre algunos lepidópteros Argentinos productores de agallas. Anales de la Sociedad Científica Argentina 82: 113–140.

[B7] Brooks SE Shorthouse JD (1988) Developmental morphology of stem galls of *Diplolepis nodulosa* (Hymenoptera: Cynipidae) and those modified by the inquiline *Periclistus pirata* (Hymenoptera: Cynipidae) on *Rosa blanda* (Rosaceae). Canadian Journal of Botany 76: 365–381.

[B8] Cabrera AL (1938) Revisión de las Anacardiáceas Austroamericanas. Revista del Museo de La Plata 2: 3–64.

[B9] Centrella ML Shaw SR (2010) A new species of phytophagous braconid *Allorhogas minimus* (Hymenoptera: Braconidae: Doryctinae) reared from fruit galls on *Miconia longifolia* (Melastomataceae) in Costa Rica. International Journal of Tropical Insect Science 30: 101–107. https://doi.org/10.1017/S1742758410000147

[B10] Centrella ML Shaw SR (2013) Three new species of gall-associated *Allorhogas* wasps from Costa Rica (Hymenoptera: Braconidae: Doryctinae). International Journal of Tropical Insect Science 33: 145–152. https://doi.org/10.1017/S1742758413000143

[B11] Chavarría L Hanson PE Marsh PM Shaw SR (2009) A phytophagous braconid, *Allorhogas conostegia* n.sp. (Hymenoptera: Braconidae), in the fruits of *Conostegia xalapensis* (Bonpl.) D. Don (Melastomataceae). Journal of Natural History 43: 2677–2689. https://doi.org/10.1080/00222930903243996

[B12] Cordeiro LP Hasenack H (2009) Cobertura vegetal atual do Rio Grande do Sul. In: Pillar VP Muller SC Castilhos ZMS Jacques AVA (Eds) Campos sulinos: Conservação e uso sustentável da biodiversidade. Ministério do Meio Ambiente, Brasília, 285–299.

[B13] Darriba D Taboada GL Doallo R Posada D (2012) jModelTest 2: more models,new heuristics and parallel computing. Nature Methods 9: 772. https://doi.org/10.1038/nmeth.2109 10.1038/nmeth.2109 PMC4594756 22847109

[B14] Davis DR (1987) Heliozelidae (Incurvarioidea). In: Stehr FW (Ed.) Immature insects. Kendall/Hunt Publishing Company, Dubuque, 354–355.

[B15] Davis DR (1998) The monotrysian Heteroneura. In: Kristensen NP (Ed.) Lepidoptera, Moths and Butterflies, vol. 1: Evolution, Systematics and Biogeography. Handbook of Zoology 4(35), Walter de Gruyter, Berlin, 65–90. https://doi.org/10.1515/9783110804744.65

[B16] Drummond AJ Ho SYW Phillips MJ Rambaut A (2006) Relaxed phylogenetics and dating with confidence. PLoS Biology 4: e88. https://doi.org/10.1371/journal.pbio.0040088 10.1371/journal.pbio.0040088 PMC1395354 16683862

[B17] Drummond AJ Rambaut A (2007) Beast: Bayesian evolutionary analysis by sampling trees. BMC Evolutionary Biology 7: 214. https://doi.org/10.1186/1471-2148-7-214 10.1186/1471-2148-7-214 PMC2247476 17996036

[B18] Ellstrand NC Elam DR (1993) Population genetic consequences of small population size: implications for plant conservation. Annual Review of Ecology and Systematics 24: 217–242. https://doi.org/10.1146/annurev.es.24.110193.001245

[B19] Excoffier L Lischer HE (2010) Arlequin suite ver 3.5: a new series of programs to perform population genetics analyses under Linux and Windows. Molecular Ecology Resources 10: 564–567. https://doi.org/10.1111/j.1755-0998.2010.02847.x 21565059 10.1111/j.1755-0998.2010.02847.x

[B20] Excoffier L Smouse PE Quattro JM (1992) Analysis of molecular variance inferred from metric distances among DNA haplotypes: Application to human mitochondrial DNA restriction data. Genetics 131: 479–491. 1644282 10.1093/genetics/131.2.479 PMC1205020

[B21] Ferreira BG Isaias RMS (2013) Developmental stem anatomy and tissue redifferentiation induced by a galling Lepidoptera on *Marcetia taxifolia* (Melastomataceae). Botany 91: 752–760. https://doi.org/10.1139/cjb-2013-0125

[B22] Fleig M (1987) Anacardiaceae. Boletim Instituto de Biociências 42: 1–72. [Flora Ilustrada do Rio Grande do Sul, 18]

[B23] Fleig M (1989) Anacardiáceas. In: Reitz R (Ed.) Flora Ilustrada Catarinense. Herbário Barbosa Rodrigues, Itajaí, 1–64.

[B24] Freitas AVL Marini-Filho OJ (Orgs) (2011) Plano de ação nacional para conservação dos lepidópteros ameaçados de extinção. Instituto Chico Mendes de Conservação da Biodiversidade. Brasilia, 124 pp. [Série Espécies Ameaçadas, 13]

[B25] Gibson JP Rice SA Stucke CM (2008) Comparison of population genetic diversity between a rare, narrowly distributed species and a common, widespread species of *Alnus* (Betulaceae). American Journal of Botany 95: 588–596. https://doi.org/10.3732/ajb.2007316 21632385 10.3732/ajb.2007316

[B26] Gozmány LA Vári L (1973) The Tineidae of the Ethiopian Region. Transvaal Museum Memoir 18: 1–238.

[B27] Guindon S Dufayard JF Lefort V Anisimova M Hordijk W Gascuel O (2010) New algorithms and methods to estimate Maximum-Likelihood phylogenies: assessing the performance of PhyML 3.0. Systematic Biology 59: 307–21. https://doi.org/10.1093/sysbio/syq010 20525638 10.1093/sysbio/syq010

[B28] Hamrick JL Godt MJW (1989) Allozyme diversity in plant species. In: Brown AHD Clegg MT Kahler AL Weir BS (Eds) Plant population genetics, breeding and genetic resources. Sinauer, Sunderland, 43–63.

[B29] Harpending HC Batzer MA Gurven M Jorde LB Rogers AR Sherry ST (1998) Genetic traces of ancient demography. Proceedings of the National Academy of Sciences 95: 1961–1967. https://doi.org/10.1073/pnas.95.4.1961 10.1073/pnas.95.4.1961 PMC19224 9465125

[B30] Hoare RJB Dugdale JS (2003) Description of the New Zealand incurvarioid *Xanadoses nielseni*, gen. n., sp. n. and placement in Cecidosidae (Lepidoptera). Invertebrate Systematics 17: 47–57. https://doi.org/10.1071/IS02024

[B31] Houard C (1933) Les zoocécidies des Plantes de l`Amérique du Sud et de l`Amérique Centrale. Librairie Scientifique Hermann et Cie, Paris, 519 pp.

[B32] Kristensen NP (2003) Skeleton and muscles: adults. In: Kristensen NP (Ed.) Lepidoptera: Moths and Butterflies, vol. 2: Morphology, Physiology, and Development. Handbook of zoology 4(36), Walter de Gruyter, Berlin, 39–131. https://doi.org/10.1515/9783110893724.39

[B33] Laroca S (1972) Notas sobre a biologia de *Hylaeus cecidonastes* Moure (Hymenoptera, Apoidea). Revista Brasileira de Biologia 32: 285–289.

[B34] Librado P Rozas J (2009) DnaSP v5: a software for comprehensive analysis ofDNA polymorphism data. Bioinformatics 25: 1451–1452. https://doi.org/10.1093/bioinformatics/btp187 19346325 10.1093/bioinformatics/btp187

[B35] Luz CLS (2011) Anacardiaceae R. Br. na flora fanerogâmica do Estado de São Paulo. Unpublished M.Sc. Thesis, São Paulo: Universidade de São Paulo.

[B36] Luz FA Gonçalves GL Moreira GRP Becker VO (2014) Three new cecidogenous species of *Palaeomystella* Fletcher (Lepidoptera, Momphidae) from the Brazilian Atlantic Rain Forest. ZooKeys 433: 97–127. https://doi.org/10.3897/zookeys.433.7379 10.3897/zookeys.433.7379 PMC4141182 25152676

[B37] Luz FA Gonçalves GL Moreira GRP Becker VO (2015) Description, molecular phylogeny, and natural history of a new kleptoparasitic species of gelechiid moth (Lepidoptera) associated with Melastomataceae galls in Brazil. Journal of Natural History 49: 1849–1875. https://doi.org/10.1080/00222933.2015.1006284

[B38] Macedo MV Monteiro RT (1989) Seed predation by a braconid wasp *Allorhogas* sp. (Hymenoptera). Journal of the New York Entomological Society 97: 358–362.

[B39] Mantel N (1967) The detection of disease clustering and a generalized regression approach. Cancer Research 27: 209–220. 6018555

[B40] Marsh PM (2002) The Doryctinae of Costa Rica (excluding the genus *Heterospilus*). Memoirs of the American Entomological Institute 70: 1–319.

[B41] Marsh PM Macedo MV Pimentel MCP (2000) Descriptions and biological notes on two new phytophagous species of the genus *Allorhogas* from Brazil (Hymenoptera: Braconidae: Doryctinae). Journal of Hymenoptera Research 9: 292–297.

[B42] Martínez JJ Altamirano A Salvo A (2011) New species of *Allorhogas* (Hymenoptera: Braconidae) reared from galls on *Lycium cestroides* Schltdl. (Solanaceae) in Argentina. Entomological Science 14: 304–308. https://doi.org/10.1111/j.1479-8298.2011.00453.x

[B43] Martínez JJ Zaldívar-Riverón A (2013) Seven new species of *Allorhogas* (Hymenoptera: Braconidae: Doryctinae) from Mexico. Revista Mexicana de Biodiversidad 84: 117–139. https://doi.org/10.7550/rmb.31955

[B44] Mey W (2007) Cecidosidae (Lepidoptera: Incurvarioidea). In: Mey W (Ed.) The Lepidoptera of the Brandberg Massif in Namibia. Esperiana Memoir 4: 31–48.

[B45] Meyrick E (1909) New South African Micro-Lepidoptera. Annals of the South African Museum 5: 349–379.

[B46] Meyrick E (1913) Description of South African Micro-Lepidoptera. 4. Annals of the Transvaal Museum 3: 267–336.

[B47] Meyrick E (1917) A jumping cocoon. Entomologist’s Monthly Magazine 53: 62.

[B48] Meyrick E (1928) Tineidae. Exotic Microlepidoptera 3: 429.

[B49] Moreira GRP Gonçalves GL Eltz RP San Blas G Davis DR (2012) Revalidation of *Oliera* Brèthes (Lepidoptera: Cecidosidae) based on a redescription of *O. argentinana* and DNA analysis of Neotropical cecidosids. Zootaxa 3557: 1–19

[B50] Moreira GRP Pollo P Brito R Gonçalves GL Vargas HÁ (2017) *Cactivalva nebularia*, gen. et sp. nov. (Lepidoptera: Gracillariidae): a new *Weinmannia* leaf miner from southern Brazil. Austral Entomology. https://doi.org/10.1111/aen.12267

[B51] Morris DC Mound LA Schwarz MP (2000) *Advenathrips inquilinus*: a new genus and species of social parasites (Thysanoptera: Phlaeothripidae). Australian Journal of Entomology 39: 53–57. https://doi.org/10.1046/j.1440-6055.2000.00146.x

[B52] Morrone JJ (2006) Biogeographic areas and transition zones of Latin America and the Caribbean Islands base on panbiogeographic and cladistics analyses of the entomofauna. Annual Review of Entomology 51: 467–94. https://doi.org/10.1146/annurev.ento.50.071803.130447 10.1146/annurev.ento.50.071803.130447 16332220

[B53] Nieves-Aldrey JL San Blas G (2015) Revision of the Neotropical genus *Eschatocerus* Mayr (Hymenoptera, Cynipidae, Eschatocerini) with biological notes and the first description of the terminal larva. Zootaxa 4012: 135–155. https://doi.org/10.11646/zootaxa.4012.1.7 26623849 10.11646/zootaxa.4012.1.7

[B54] van Nieukerken EJ Kaila L Kitching IJ Kristensen NP Lees DC Minet J Mitter C Mutanen M Regier JC Simonsen TJ Wahlberg N Yen S-H Zahiri R Adamski D Baixeras J Bartsch D Bengtsson BÅ Brown JW Bucheli SR Davis DR De Prins J De Prins W Epstein ME Gentili-Poole P Gielis C Hättenschwiler P Hausmann A Holloway JD Kallies A Karshol O Kawahara A Koster JC Kozlov MV Lafontaine JD Lamas G Landry J-F Lee S Nuss M Park K-T Penz C Rota J Schmidt BC Schintlmeister A Sohn JC Solis MA Tarmann GM Warren AD Weller S Yakovlev VR Zolotuhin VV Zwick A (2011) Order Lepidoptera. In: Zhang Z-Q (Ed.) Animal biodiversity: An outline of higher-level classification and survey of taxonomic richness. Zootaxa 3148: 212–221.

[B55] Noort S van Stone GN Whitehead VB Nieves-Aldrey J-L (2007) Biology of *Rhoophilus loewi* (Hymenoptera: Cynipoidea: Cynipidae), with implications for the evolution of inquilinism in gall wasps. Biological Journal of the Linnean Society 90: 153–172. https://doi.org/10.1111/j.1095-8312.2007.00719.x

[B56] Overbeck GE Muller SC Fidelisa A Fadenhauer JP Pillar VP Blanco CC Boldrini II Both R Forneck ED (2007) Brazil’s neglected biome: The South Brazilian Campos. Perspectives in Plant Ecology, Evolution and Systematic 9: 101–116. https://doi.org/10.1016/j.ppees.2007.07.005

[B57] Pellmyr O Leebens-Mack JH (1999) Forty million years of mutualism: evidence for Eocene origin of yucca–yucca moth association. Proceedings National Academy of Science 96: 9178–9183. https://doi.org/10.1073/pnas.96.16.9178 10.1073/pnas.96.16.9178 PMC17753 10430916

[B58] Penteado-Dias AM Carvalho FM (2008) New species of Hymenoptera associated with galls on *Calliandra brevipes* Benth. (Fabaceae, Mimosoidea) in Brazil. Revista Brasileira de Entomologia 52: 305–310. https://doi.org/10.1590/S0085-56262008000300001

[B59] Raman A (1994) Adaptation integration between gall-inducing insects and their host plants. In: Anathakrishnan TN (Ed.) Functional dynamics of phytophagous insects. Science Publishers, Lebanon, 249–275.

[B60] Rambaut A (2009) Molecular evolution, phylogenetics and epidemiology: FigTree. http://tree.bio.ed.ac.uk/software/figtree/

[B61] Regier JC Mitter C Kristensen NP Davis DR van Nieukerken EJ Rota J Simonsen TJ Mitter KT Kawahara AY Yen S-H Cummings MP Zwick A (2015) A molecular phylogeny for the oldest (nonditrysian) lineages of extant Lepidoptera, with implications for classification, comparative morphology and life-history evolution. Systematic Entomology 40: 671–704. https://doi.org/10.1111/syen.12129

[B62] Rogers AR Harpending H (1992) Population growth makes waves in the distribution of pairwise genetic differences. Molecular Biology and Evolution 9: 552–569. 1316531 10.1093/oxfordjournals.molbev.a040727

[B63] San Blas G Davis DR (2013) Redescription of *Dicranoses capsulife*x Kieffer and Jörgensen (Lepidoptera: Cecidosidae) with description of the immature stages and biology. Zootaxa 3682: 371–384. https://doi.org/10.11646/zootaxa.3682.2.9 25243292 10.11646/zootaxa.3682.2.9

[B64] Sáiz F Núñez C (1997) Estudio ecológico de las cecidias del género *Schinus*, especialmente las de hoja y de rama de *S. polygamus* y *Schinus latifolius* (Anacardiaceae), en Chile Central. Acta Entomológica Chilena 21: 39–59.

[B65] Schneider S Excoffier L (1999) Estimation of past demographic parameters from the distribution of pairwise differences when the mutation rates vary among sites: Application to human mitochondrial DNA. Genetics 152: 1079–1089. 10388826 10.1093/genetics/152.3.1079 PMC1460660

[B66] Schwarz GE (1978) Estimating the dimension of a model. Annals of Statistics 6: 461–464. https://doi.org/10.1214/aos/1176344136

[B67] Slatkin M Hudson RR (1991) Pairwise comparisons of mitochondrial DNA sequences in stable and exponentially growing populations. Genetics 129: 555–562. 1743491 10.1093/genetics/129.2.555 PMC1204643

[B68] Stone GN Schönrogge K (2003) The adaptive significance of insect gall morpholoy. Trends in Ecology and Evolution 18: 512–522. https://doi.org/10.1016/S0169-5347(03)00247-7

[B69] Tajima F (1989) Statistical method for testing the neutral mutation hypothesis by DNA polymorphism. Genetics 123: 585–595. 2513255 10.1093/genetics/123.3.585 PMC1203831

[B70] Tamura K Stecher G Peterson D Filipski A Kumar S (2013) MEGA6: molecular Evolutionary Genetics Analysis version 6.0. Molecular Biology and Evolution 30: 2725–2729. https://doi.org/10.1093/molbev/mst197 24132122 10.1093/molbev/mst197 PMC3840312

[B71] Tavares JS (1909) Contributo prima ad cognitionem Cecídologiae Braziliae. Broteria 8: 5–29.

[B72] Tavares JS (1917) As cecídeas do Brazil que se criam nas plantas da familia das Melastomataceae. Broteria 15: 18–49.

[B73] Tauber MJ Tauber CA Masaki S (1986) Seasonal adaptations of insects. Oxford University Press, New York, 426 pp.

[B74] Vecchi C Menezes NL Oliveira DC Ferreira BG Isaias RMS (2013) The redifferentiation of nutritive cells in galls induced by Lepidoptera on *Tibouchina pulchra* (Cham.) Cogn. reveals predefined patterns of plant development. Protoplasma [Online] https://doi.org/10.1007/s00709-013-0519-6 10.1007/s00709-013-0519-6 23779213

[B75] Walhberg N Wheat CW Peña C (2013) Timing and patterns in the taxonomic diversification of Lepidoptera (butterflies and moths). PLoS ONE 8: e80875. https://doi.org/10.1371/journal.pone.0080875 10.1371/journal.pone.0080875 PMC3839996 24282557

[B76] Wille J (1926) *Cecidoses eremita* Curt. Und ihre Galle an *Schinus dependens* Ortega. Zeitschrift für Morphologie und Ökologie der Tiere 7: 1–101. https://doi.org/10.1007/BF00540718

[B77] Yukawa J (2000) Synchronization of gallers with host plant phenology. Population Ecology 42: 105–113. https://doi.org/10.1007/PL00011989

