# Supplementary material for: Cecidonius pampeanus, gen. et sp. n.: an overlooked and rare, new gall-inducing micromoth associated with Schinus in southern Brazil (Lepidoptera, Cecidosidae)
Source: Zookeys. 2017 Sep 4;(695):37–74. doi: 10.3897/zookeys.695.13320 (PMC5673834; doi:10.3897/zookeys.695.13320)
Supplement: Supplementary material 1 — Table S1. [file zookeys-695-037-s001.docx]

Table S1. Primers and conditions used in polymerase chain reaction (PCR) to amplify COI, 16S and Wg genes.

| Locus | Abbreviation | Fragment length (bp) | Primers (F/R) |  | PCR | |
| --- | --- | --- | --- | --- | --- | --- |
|  |  |  |  |  | Reaction | annealing temperature |
| Cytochrome oxidase subunit I | COI | ca.1420 | K698 (F)^a^ | TACAATTTATCGCCTAAACTTCAGCC | 94°C, 5 min; 30 cycles of 94°C for 15 s, N°C for 30 s, and 72°C for 30 s; 72°C, 5 min | 48°C |
|  |  |  | Nancy (R)^a^ | TACAATTTATCGCCTAAACTTCAGCC |  | 48°C |
|  |  |  | Jerry (F)^a^ | CAACATTTATTTTGATTTTTTGG |  | 48°C |
|  |  |  | Pat II (R)^a^ | TCCATTACATATAATCTGCCATATTAG |  | 48°C |
| 16S ribosomal RNA | 16S | ca.470 | 16Sar1(F)^b^ | CCCGCCTGTTTATCAAAAACA |  | 47°C |
|  |  |  | Ins16Sar (R)^b^ | CCCTCCGGTTTGAACTCAGAT |  | 47°C |
| Wingless | Wg | ca. 380 | LepWg1(F)^c^ | GARTGYAARTGYCAYGGYATGTCTGG |  | 45°C |
|  |  |  | LepWg2a (R)^c^ | ACT NCGCARCACCARTGGAATGTRCA |  | 45°C |

^a^ Caterino and Sperling (1999); ^b^ Palumbi (1996); ^c^ Brower & DeSalle (1998)
