# Supplementary material for: Cecidonius pampeanus, gen. et sp. n.: an overlooked and rare, new gall-inducing micromoth associated with Schinus in southern Brazil (Lepidoptera, Cecidosidae)
Source: Zookeys. 2017 Sep 4;(695):37–74. doi: 10.3897/zookeys.695.13320 (PMC5673834; doi:10.3897/zookeys.695.13320)
Supplement: Supplementary material 2 — Table S2. [file zookeys-695-037-s002.docx]

Table S2. Analysis of Molecular Variance (AMOVA) using ɸ-statistics based on cytochrome oxidase subunit I mitochondrial sequences for groups of *C. pampeanus* defined according to different dispersal barriers.

| Group definition | Populations | Va | Vb | Vc | FST | FSC | FCT | *P* |
| --- | --- | --- | --- | --- | --- | --- | --- | --- |
| Jacuí River acting as barrier | [P1+P3] and [P2+P4+P5+P6+P7+P8+P9+P10] | 2.9698 (46.45%) | 3.2674  (51%) | 0.1633 (2.55%) | 0.9744 | 0.9489 | 0.5873 | <0.001 |
| Major network distance | [P1+P2+P3] and [P4+P5+P6+P7+P8+P9+P10] | 4.5520 (58.73%) | 3.0348  (39.16%) | 0.1633 (2.11%) | 0.9789 | 0.9523 | 0.4639 | <0.001 |
