# Supplementary material for: Cecidonius pampeanus, gen. et sp. n.: an overlooked and rare, new gall-inducing micromoth associated with Schinus in southern Brazil (Lepidoptera, Cecidosidae)
Source: Zookeys. 2017 Sep 4;(695):37–74. doi: 10.3897/zookeys.695.13320 (PMC5673834; doi:10.3897/zookeys.695.13320)
Supplement: Supplementary material 3 — Table S3. [file zookeys-695-037-s003.docx]

Table S3. Specimens used in this study for phylogenetic reconstruction and genetic structure analysis of *Cecidonius pampeanus*.

|  |  |  |  |  |  | **Genbank (accession number)** | | |
| --- | --- | --- | --- | --- | --- | --- | --- | --- |
| **Family** | **Genus** | **Species** | **Pop.** | **Vouchers*** | **Haplotype** | **COI** | **16S** | **Wg** |
| Cecidosidae |  |  |  |  |  |  |  |  |
|  | *Cecidonius* | *pampeanus* sp.n. | 1 | LMCI 75-1 to 75-5 | H13 | XXXXX | - | - |
|  |  |  | 2 | LMCI 39-14 to 39-19 | H12 | XXXXX | - | - |
|  |  |  | 3 | LMCI 77-13 to 77-18 | H14 | XXXXX | - | - |
|  |  |  | 4 | LMCI 4-47, 4-48, 16-24 to 16-27 | H2 | XXXXX | - | - |
|  |  |  | 5 | LMCI 1-1, 1-3,1-6 to 1-9 | H1 | XXXXX | - | - |
|  |  |  | 6 | LMCI 35-17, 35-18, 35-20, 35-22, 35-24, 35-25 | H1, H5* | XXXXX | - | - |
|  |  |  | 7 | LMCI 36-14 to 36-16, 36-20, 36-21 | H6, H7 | XXXXX/ XXXXX | - | - |
|  |  |  | 8 | LMCI 18-21 to 18-26 | H3, H4 | XXXXX/ XXXXX | - | - |
|  |  |  | 9 | LMCI 37-13, 37-15, 37-18, 37-19, 37-31 | H8*, H9 | XXXXX | - | - |
|  |  |  | 10 | LMCI 38-16 to 38-21 | H9, H10*, H11* | XXXXX/ XXXXX | - | - |
|  | *Cecidonius* | sp. | - | LMCI 14-72, 14-74 | - | XXXXX | XXXXX | XXXXX |
|  | Cecidosidae | sp. | - | LMCI 163-14B, 233-6 | - | XXXXX | XXXXX | XXXXX |
|  | *Cecidoses* | *eremita* | - | LMCI 163-1A, 16-1 | - | XXXXX | XXXXX | XXXXX |
|  | *Dicranoses* | *congregatella* | - | LMCI 3-1 | - | XXXXX | XXXXX | XXXXX |
|  | *Eucecidoses* | *minutanus* | - | LMCI 163-21 | - | XXXXX | XXXXX | XXXXX |
|  | *Oliera* | *argentinana* | - | LMCI 6-11 | - | XXXXX | XXXXX | XXXXX |
|  | *Scyrotis* | sp. | - | LMCI 228-1 | - | XXXXX | - | - |
|  |  | *granosa* | - | LMCI 228-2 | - | XXXXX | - | - |
| Prodoxidae |  |  |  |  |  |  |  |  |
|  | *Greya* | *enchrisa* | - | - | - | EU884123 | - | - |
|  | *Tegeticula* | *antithetica* | - | - | - | EU585222 | - | - |

*Larvae preserved in 100% ethanol at -20 ^o^C, dissected from galls induced on *S. weinmannifolius* plants, collected from RS localities, preserved in the tissue collection of Laboratório de Morfologia e Comportamento de Insetos (LMCI), as follows: **Pop. 1 (= Population 1)** > LMCI 75-1 to 29 (n = 29), Capão do Valo, Cachoeira do Sul municipality, 29°54'10"S, 52°25'11"W, 86m, 02.V.2009, S.A.L. Bordignon leg.; **Pop. 2** > LMCI 39-14 to 32 (n = 20), Belvedere, Encruzilhada do Sul municipality, 30°22'10"S, 52°25'46"W, 210m, 18.V.2008, G.R.P. Moreira leg.; **Pop. 3** > LMCI 76-12 to 28 (n = 17), Santo Amaro, General Camara municipality, 29°55'49"S, 51°53'24"W, 65m, 8.V.2009, G.R.P. Moreira, S.A.L. Bordignon & G. Von Poser leg.; **Pop. 4** > LMCI 16-24 to 43 (n = 20), Morro São Maximiano, Eldorado do Sul municipality, 30°10'46''S, 50°23'21''W, 14.VIII.2007, 56m, G.R.P. Moreira & G.L. Gonçalves leg.; **Pop. 5** > LMCI 1-1 to 10 (n = 10), Morro do Osso, Porto Alegre municipality, 30°07'05''S, 51°14'37''W, 112m, 20.VII.2007, G.R.P. Moreira & R.P. Eltz leg.; **Pop. 6** > LMCI 35-16 to 35 (n = 20), Morro da Tapera, Porto Alegre municipality, 30°06'53"S, 51°11'47"W, 156m, 13.V.2008, G.R.P. Moreira & G. Buss leg.; **Pop. 7** > LMCI 36-12 to 31 (n = 20), Morro Santana, 30°03'12''S, 51°07'14''W, 295m, Porto Alegre municipality, 13.V.2008, G.R.P. Moreira & L.R. Jorge, leg.; **Pop. 8** > LMCI 18-21 to 40 (n = 20), Morro da Extrema, Porto Alegre municipality, 30°11'44´´S, 51°02'22" W, 161, 18.III.2008, G.R.P. Moreira & G. Buss leg.; **Pop. 9** > LMCI 37-13 to 32 (n = 20), Lombas de Viamão, Viamão municipality, 30^◦^04'30''S; 50^◦^41'08''W, 49m, 14.V.2008, G.R.P. Moreira & S.A.L.Bordignon leg.; **Pop. 10** > LMCI 38-15 to 34 (n = 20), Lombas de Santo Antônio, Santo Antônio da Patrulha municipality, 30^◦^01'36''S, 50^◦^36'49''W, 78m, 14.V.2008, G.R.P. Moreira & S.A.L Bordignon leg.

Additional larvae preserved in 100% ethanol at -20 ^o^C, used for DNA extraction for comparison: from ***Cecidonius* sp**., dissected from galls induced on *Schinus therebinthifolius* Raddi, LMCI 14-71 to 74 (n = 4), Parque Passaúna, Campo Comprido municipality, Paraná State, Brazil, 25°27'35"S, 49°22'50"W, 905m, 22.II.2008, G.R.P. Moreira, O.S. Ribas, E. Carneiro & L. Beltrami legs.; from **Cecidosidae sp**., LMCI 163-14 (n = 3), Rungue/Tiltil, Chile, 12.10.2011, G.San Blas leg.; LMCI 233-6 (n =1), Cuesta La Dormida, 28.11.2013, H.A Vargas & G.R.P. Moreira legs.; from ***Cecidoses eremita***, LMCI16-1 to 20 (n=20), Morro Maximiano, Eldorado do Sul municipality, RS, Brazil, 14.03.2007, G. R.P.Moreira & G.L. Gonçalves legs.; from ***Dicranoses congregatella***, LMCI 3-1 to 10 (n =10), Rincão da Ronda, Canguçú municipality, RS, Brazil, 20.07.2007, G.R.P. Moreira leg.; from ***Eucecidoses minutanus***, LMC163-21(n =4), Las Heras, Mendoza, Argentina, 26.10.2011, G. San Blas & G.R.P. Moreira legs.; from ***Oliera argentinana***, LMC 6-1 to 15 (n=15), Rincão da Ronda, Canguçú municipality, RS, Brazil, 15.10.2007, G.R.P. Moreira leg.; LMCI 163-1 (n = 2), Luján de Cuyo, Mendoza, Argentina, 14.11.2009. G. San Blas leg.;.

Also used for DNa extraction for comparison, three pine-dried specimens from South Africa, all collected by Wolfram Mey: ***Scyrotis* sp.**, female, RSA, Eastern Cape, Asante Sana, leg. 2012 (LMCI 228-1); ***Scyrotis granosa*** Meyrick, male, RSA, Tsitsikamma, 2011 (LMCI 228-2); ***Scyrotis pulleni*** Mey, paratype, RSA, Mpumalanga, 2005 (LMCI228-3) that failured regarding DNA extraction.
