# Supplementary material for: Cecidonius pampeanus, gen. et sp. n.: an overlooked and rare, new gall-inducing micromoth associated with Schinus in southern Brazil (Lepidoptera, Cecidosidae)
Source: Zookeys. 2017 Sep 4;(695):37–74. doi: 10.3897/zookeys.695.13320 (PMC5673834; doi:10.3897/zookeys.695.13320)
Supplement: Supplementary material 4 — Figure S1. [file zookeys-695-037-s004.docx]

Figure S1. Graphs depicting the results of the mismatch distribution analysis for the total of *C. pampeanus* samples and populations alone (P6 to P10). The analysis was performed with 1420 bp of COI sequences (excluding all sites with missing information or gaps).


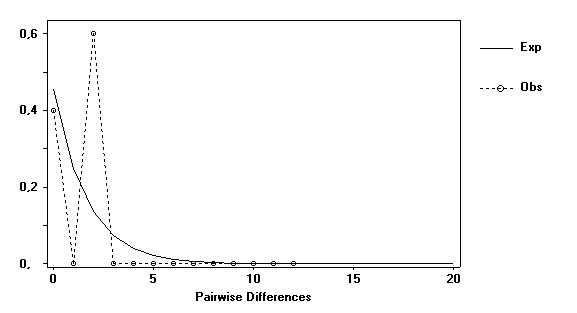

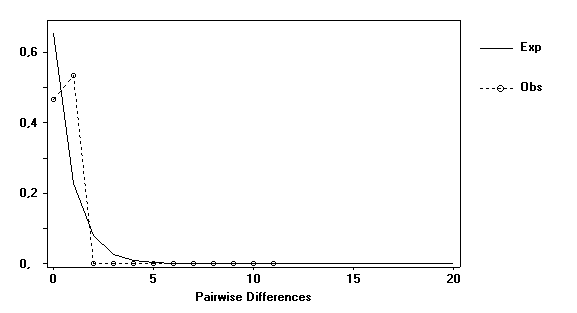

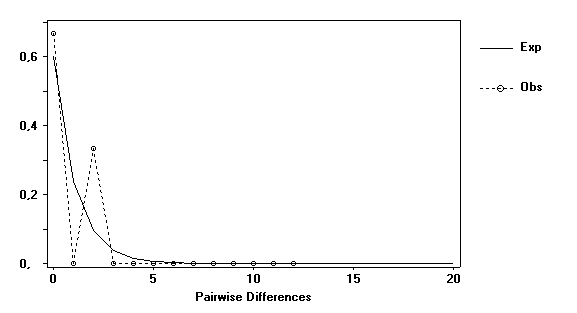

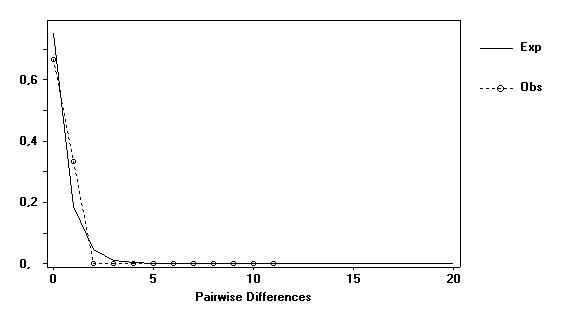

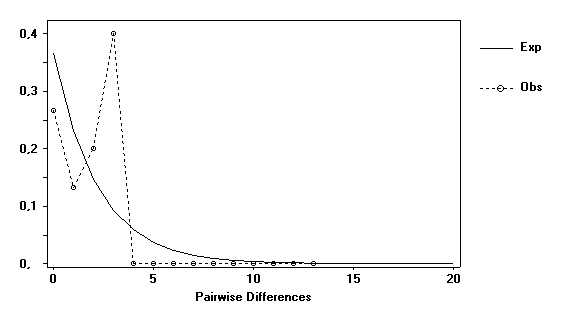

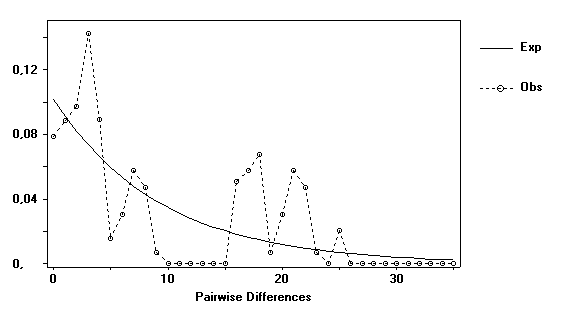


*Cecidonius pampeanus*

P8

P9

P10

P6

P7
