# Supplementary material for: Cecidonius pampeanus, gen. et sp. n.: an overlooked and rare, new gall-inducing micromoth associated with Schinus in southern Brazil (Lepidoptera, Cecidosidae)
Source: Zookeys. 2017 Sep 4;(695):37–74. doi: 10.3897/zookeys.695.13320 (PMC5673834; doi:10.3897/zookeys.695.13320)
Supplement: Supplementary material 5 — Figure S2. [file zookeys-695-037-s005.docx]

**Figure S2.** Neighbor-Joining tree of *Cecidonius pampeanus* with the evolutionary distances computed using the Kimura 2-parameter method based on 1.6 Kb of cytochrome oxidase sequences. The analysis involved 60 individuals from 10 populations.
